# Devising Bone Molecular Models at the Nanoscale: From Usual Mineralized Collagen Fibrils to the First Bone Fibers Including Hydroxyapatite in the Extra-Fibrillar Volume

**DOI:** 10.3390/ma15062274

**Published:** 2022-03-19

**Authors:** Amadeus C. S. Alcântara, Levi C. Felix, Douglas S. Galvão, Paulo Sollero, Munir S. Skaf

**Affiliations:** 1 Department of Computational Mechanics, School of Mechanical Engineering, University of Campinas—UNICAMP, Campinas 13083-860, SP, Brazil; amadeus.alcantara@fem.unicamp.br (A.C.S.A.); sollero@fem.unicamp.br (P.S.); 2 Center for Computing in Engineering & Sciences, CCES, University of Campinas—UNICAMP, Campinas 13083-861, SP, Brazil; felixce@ifi.unicamp.br (L.C.F.); galvao@ifi.unicamp.br (D.S.G.); 3 Department of Applied Physics, Gleb Wataghin Institute of Physics, University of Campinas—UNICAMP, Campinas 13083-859, SP, Brazil; 4 Institute of Chemistry, University of Campinas—UNICAMP, Campinas 13083-970, SP, Brazil

**Keywords:** bone nanoscale model, mineralized collagen fibril, collagen fiber, hydroxyapatite, extra-fibrillar volume, molecular dynamics, bone elastic properties

## Abstract

At the molecular scale, bone is mainly constituted of type-I collagen, hydroxyapatite, and water. Different fractions of these constituents compose different composite materials that exhibit different mechanical properties at the nanoscale, where the bone is characterized as a fiber, i.e., a bundle of mineralized collagen fibrils surrounded by water and hydroxyapatite in the extra-fibrillar volume. The literature presents only models that resemble mineralized collagen fibrils, including hydroxyapatite in the intra-fibrillar volume only, and lacks a detailed prescription on how to devise such models. Here, we present all-atom bone molecular models at the nanoscale, which, differently from previous bone models, include hydroxyapatite both in the intra-fibrillar volume and in the extra-fibrillar volume, resembling fibers in bones. Our main goal is to provide a detailed prescription on how to devise such models with different fractions of the constituents, and for that reason, we have made step-by-step scripts and files for reproducing these models available. To validate the models, we assessed their elastic properties by performing molecular dynamics simulations that resemble tensile tests, and compared the computed values against the literature (both experimental and computational results). Our results corroborate previous findings, as Young’s Modulus values increase with higher fractions of hydroxyapatite, revealing all-atom bone models that include hydroxyapatite in both the intra-fibrillar volume and in the extra-fibrillar volume as a path towards realistic bone modeling at the nanoscale.

## 1. Introduction

If current preventive diagnosis techniques remain unimproved, aging-related bone diseases, such as osteoporosis and their subsequent bone fractures are expected to overload health care systems worldwide [1]. Understanding the mechanical properties of bones at each length scale is essential to improving such techniques. Computer simulations allow the investigation of mechanical properties at all length scales by combining mathematical, physical, engineering, and biological concepts [2]. Furthermore, the more realistic they are, the more reliable such preventive diagnosis techniques become.

Bones are patient-specific and exhibit a multiscale structure [2,3,4]. This means that a bone fragment from a given individual exhibits a complex network of different physical structures and mechanical properties down to the molecular scale, where fracture ultimately originates. Thus, the best-achievable simulations must seek to: (1) consider bones as patient-specific by devising different models with different fractions of the constituents, testing several specimens of a statistical population, or by extracting geometry and mechanical properties directly from the targeted bone, e.g., from computed tomography; (2) consider the multiscale nature of bone by modeling and coupling several length scales, or by devising models that directly include information from other length scales.

Several works performing molecular dynamics (MD) simulations of the bone structure have tried to comply with these two points, as shown by recent reviews [2,3,5]. Especially after Ref. [6] made available the first fibrillar structure of type I collagen, i.e., the structure of bone at the sub-nanoscale [2], MD simulations were carried out to study the heterogeneous nature of collagen [7,8], the orientation and chemical processes of its structure [9,10], and its mechanical properties [11]. Subsequently, hydroxyapatite crystals were included in the models based on the fibrillar structure provided by Ref. [6] for further investigations, especially for the mechanical properties [12,13,14,15,16,17]. To date, hydroxyapatite has been included solely within fibrils, in the intra-fibrillar volume (IFV). Yet, as several experiments have shown, higher concentrations of hydroxyapatite are indeed found surrounding the fibrils in the extra-fibrillar volume (EFV) [18,19,20,21,22,23], which is also labeled as the extra-fibrillar matrix.

**Figure 1:**
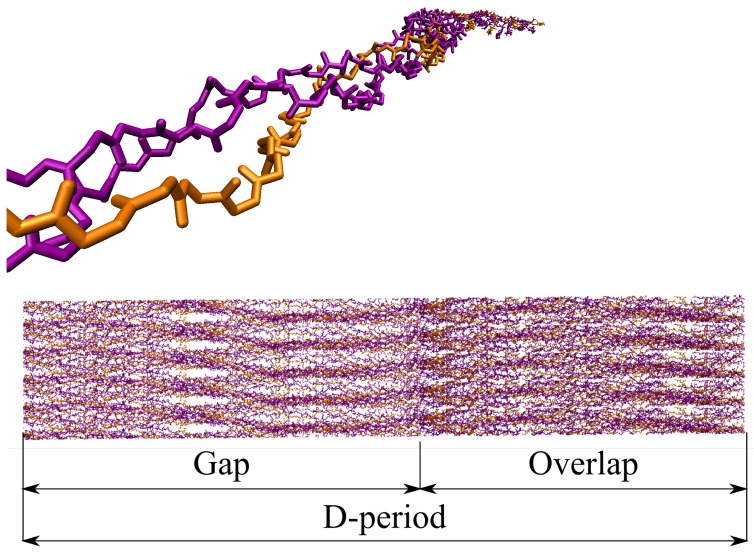
Structural representation of the backbone of a single molecule (**top**) and fibril (**bottom**) of the type I CLG. Chains A and C (alpha-1) are indicated in the purple color, and Chain B (alpha-2) in orange one.

Understanding the mechanical properties of bones and the molecular aspects that underlie their behavior at small non-continuous length scales constitutes an open field of research and requires substantial further endeavors. This work aims to contribute to the field by: (1) detailing the process of modeling all-atom bone collagen fibrils (subnanoscale [2]) and, for the first time, fibers (nanoscale [2]); (2) investigating the mechanical properties of bone at the nanoscale to validate the model. This paper details how all-atom models that resemble the structure of fibers in bones can be devised, and how they can be subjected to nanoscale traction tests to assess their Young’s Modulus values. The models consist of a bundle of mineralized collagen fibrils surrounded by hydroxyapatite in the EFV, similar to the experiments presented in Ref. [20] Figure 4 (reproduced in Ref. [24] Figure 1), and Ref. [18] Figure 8. All files and scripts used to devise the described models are available in the Supplementary Materials.

### 1.1. Reading This Paper–Textual Organization and Notation

This paper covers a multidisciplinary topic, which may attract the attention of researchers from different fields, including biology, medicine, physics, chemistry, and engineering. Thus, inspired by Ref. [2], four extra text environments were used to increase the paper readability:

**Definition:**  Non-mathematical definitions that may be differently understood by specialists from different fields;

**Highlight:**  A statement that plays a major role in the interpretations and discussions of the results;

**Open Issue:**  Issues and problems not clearly defined or not yet completely solved within the surveyed literature;

**Remark:**  Relevant notes.

The appendices contain detailed information about the modeling process. Readers seeking to reproduce the models are advised to read the main text along with the appendices.

### 1.2. The Multiscale Structure of Bone: From the Molecular Scale to the Nanoscale

At the molecular scale, bone is a unique and complex composite material mainly composed of type I collagen (CLG), hydroxyapatite (HA) Ca_10_(PO_4_)_6_(OH)_2_, and water (H_2_O) [2,25,26,27,28]. Different fractions of these constituents lead to different mechanical properties of the material; bones with a lower concentration of HA usually display lower stiffness, and vice versa [12,29].

- Reference values for their volume fractions are: 33–43% mineral material (mainly HA), 32–44% organic material (mainly CLG), and 15–25% H${}_{2}$O [23,30].
- Reference values for their mass fractions are: 60–65% mineral material (mainly HA), 25–30% organic material (mainly CLG), and 10% H${}_{2}$O [22,30,31,32].

A single CLG molecule, i.e., a tropocollagen, is a helical structure consisting of three (two alpha-1 and one alpha-2) left-handed polypeptide chains coiled around each other to form a right-handed superhelix; see Figure 1. A polypeptide chain consists of a sequence of amino acids covalently linked by peptide bonds. An alpha-amino acid (labeled here as simply amino acid) is an organic compound that contains an amino group (NH${}_{2}$), a carboxyl group (COOH), and an R group, and is also known as a *side chain*. A peptide bond is the CO–NH chemical covalent bond formed between two molecules when the C of the carboxyl group of one molecule reacts with the N of the amino group of the other molecule, releasing a molecule of H${}_{2}$O.

The amino and carboxyl groups are standard parts of amino acids. The R group can vary among amino acids. Thus, it is the R group that defines the type of amino acid. Type I CLG displays polypeptide chains that consist mostly of GLY-X-Y. This means that one in three amino acids is a *glycine*. The most common amino acids present in the X and Y positions are *proline* (PRO) and *hydroxyproline* (HYP), respectively. Prolines at the third position of the tripeptide repeating unit GLY-X-Y tend to be hydroxylated, turning into hydroxyproline.

At the sub-nanoscale, a collection of axially connected CLG molecules arranged side by side forms a collagen fibril (CLGf); see Figure 1. A CLGf is labeled a mineralized collagen fibril (mCLGf) when there are HA crystals between the CLG molecules, mostly in their gap zones. Although denser than gap zones, mCLGf overlap zones can also exhibit HA molecules. In short, an mCLGf is a CLG fibril filled with HA in the IFV, the IFV being composed of CLG fibrils, gap zones, and overlap zones. Furthermore, a bundle of fibrils forms a fiber. At the nanoscale, bone can be described as a fiber built by a combination of wet CLGfs and mCLGfs with surrounding $H_{2}O$ and HA crystals in the EFV.

**Remark** **1** (Bone Length Scales). *The multiple length scales of bone are not equally structured and represented in the literature. The structure presented by Ref. [2], Figure 4, Section 13 is adopted here. For further reading regarding bone multiscale characteristics, see Refs. [2,4,30,33].*

In brief, from the molecular scale up to the nanoscale, bone is composed of a large number of interacting molecules. Each molecule comprises several atoms participating in interatomic bonds. Assuming that modeling each atom as a solid particle and each bond as an elastic spring is accurate enough, the molecular/nanoscale domain is defined as a gathering of discrete particles, i.e., a non-continuum, which is mostly studied through MD simulations.

## 2. Materials and Methods: Devising Bone at the Nanoscale

### 2.1. Devising the Simulation Box

Here, a step-by-step description is given of how models that resemble fibers in bones can be devised.

**Figure 2:**
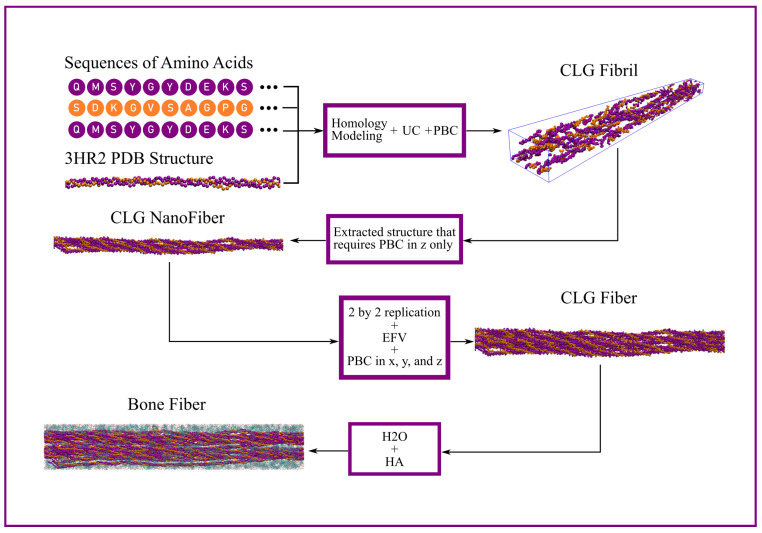
Schematic view of the modeling of a structure that resembles fibers in bones.

First, starting from the sequence of amino acids and an available fibrillar structure, the CLG Fibril model was devised through homology modeling. Then, a structure of the CLG fibril that requires Periodic Boundary Conditions (PBCs) only along the z direction was extracted and labeled CLG NanoFiber. When the latter is replicated along the x and y directions, surrounded by an EFV, and subjected to PBCs in the x, y, and z directions, the newly devised model is labeled CLG Fiber. Finally, adding $H_{2}O$ and HA both in the EFV and IFV of the CLG Fiber gives origin to the Bone Fiber model. See Figure 2 for a schematic view of this modeling process, described in detail throughout this section.

#### 2.1.1. CLG Fibril

     Different from most proteins, CLG is not found isolated and fully solvated in bones, and it does not completely fold to perform a specific function. It is the association of CLGs under *physiological conditions* into fibrils and, consequently, fibers, which confer CLG-based tissues with their remarkable macroscale mechanical properties, such as high tensile strength. Thus, it is crucial to reproduce the fibrillar and fiber structure in MD simulations when studying the CLG mechanical properties.

**Definition** **1** (Physiological Conditions). *In biochemistry, reactions are usually studied under physiological conditions, that is, an electrically neutral aqueous solution at 1 atm pressure, ~37 °C temperature, 0.16 mol/L salt concentration ($Na^{+}$ and $Cl^{-}$ ions), ”enantiomer specific”, and a specific pH.*

To date, only the amino acid sequence, i.e., the primary protein structure, of human type I CLG has been fully determined. This can be found at the Universal Protein Resource (UniProt) website [34] under the codes COL1A1_human (P02452) and COL1A2_human (P08123) for the alpha-1 and alpha-2 chains, respectively. However, to perform MD simulations, the spatial position of every atom, i.e., at least the tertiary protein structure, is required. Several high-resolution structures such as 1WZB [35], which periodically reproduces a common amino acid pattern of the CLG, can be found in the Protein Data Bank (PDB) [36] and can be used as approximations of the type I CLG human structure. However, as mentioned before, it is crucial to reproduce the fibrillar and fiber structure, i.e., the quaternary protein structure, when studying the CLG mechanical properties.

**Definition** **2** (High-Resolution and Low-Resolution Protein Structures). *Low-resolution structures usually contain only the position of the alpha carbons (CA). All other atomic positions, e.g., side-chain atoms, must be inferred. High-resolution structures usually contain the position of every non-hydrogen atom.*

Unfortunately, there is no experimentally determined molecular structure of the quaternary protein structure of the human type I CLG available in the PDB. An alternative for modeling the human type I CLG structure is *homology modeling*.

**Definition** **3** (Homology Modeling). *Also labeled comparative modeling of protein 3D structures, homology modeling is a procedure that produces a previously unknown 3D protein structure by associating an amino acid sequence (labeled the target) with a known experimentally determined 3D atomic-resolution structure (labeled the template) of a homologous sequence. Two amino acid sequences are considered homologous when they are very similar, e.g., they display a high sequence identity value, meaning that they share a common evolutionary ancestry. Homologous sequences display similar structures and, frequently, similar functions [37].*

The PDB structure 3HR2 [6], an experimentally determined low-resolution crystal structure for type I CLG of rat tail tendons, is, to our knowledge, the only structure available in the PDB that encompasses the fibrillar structure of type I CLG. It reproduces the fibrillar structure as a crystal, with a unit cell (UC) that is periodically replicated along the x, y, and z directions.

When aligned, the type I CLG amino acid sequences of the human—Uniprot P02452 and P08123—and rat—PDB 3HR2—exhibit sequence identity above 90%, indicating that they are highly homologous. Hence, they are appropriate for comparative structural modeling. If the 3HR2 structure were a high-resolution structure, it could be directly used for the MD simulations proposed here. However, since it contains only the positions of the CA atoms of the amino acids, the position of the non-CA atoms must be inferred. Homology modeling allows the inference of the positions of the non-CA atoms.

*MODELLER 9.25* [38] was used to build a homology model that correlates the human amino acids sequences—Uniprot P02452 and P08123—with the rat fibrillar CLG structure—PDB 3HR2. In Appendix A, the necessary steps to build this model are described. All the necessary files and scripts for its reproduction together with further details are also provided in the Supplementary Materials.

**Figure 3:**
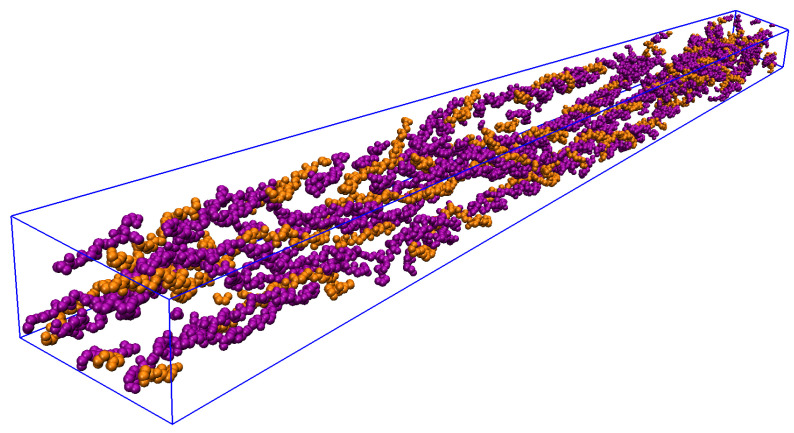
CLG Fibril within UC; snapshot from VMD viewer.

**Figure 4:**
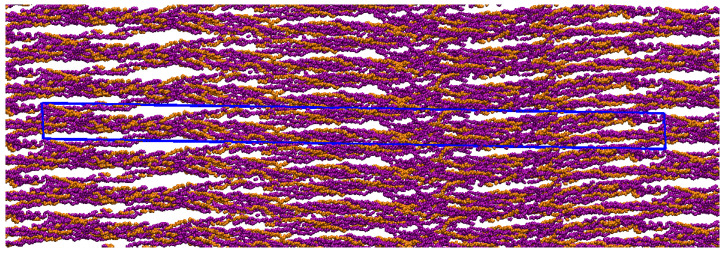
CLG Fibril periodically replicated in space; snapshot from VMD viewer.

When compressed in the crystal-like triclinic UC determined by Ref. [6] (*a* = 39.970 Å; *b* = 26.950 Å; *c* = 677.900 Å; α = 89.24${}^{{}^{\circ}}$; β = 94.59${}^{{}^{\circ}}$; γ = 105.58${}^{{}^{\circ}}$; see Figure 3), and periodically replicated in space through periodic boundary conditions (PBCs) (see Figure 4), the built homology model reproduces the type I CLG fibrillar structure experimentally determined by Ref. [6]. This new model is labeled CLG Fibril throughout this paper. It can be devised by performing three steps:

1. Importing the homology model, built as described in Appendix A, into VMD [39,40] (http://www.ks.uiuc.edu/Research/vmd/, accessed on 30 January 2022). The H atoms can be kept or not. The models built here did not keep the H atoms, since they can be added later using the VMD software when generating a PSF file, as described in Appendix C;
2. Setting triclinic UC dimensions using the “pbc set {39.970 26.950 677.900 89.24 94.59 105.58}” command of the VMD PBCTools Plugin in the VMD TkConsole;
3. Wrapping all atoms into the defined UC using the ”pbc wrap” command of the VMD PBC Tools Plugin in the VMD TkConsole.

Models such as the CLG Fibril, which combine the human amino acids sequences with the rat fibrillar CLG structure, have been previously reported; see Refs. [8,9,10,11,12,13,17,41,42]. Ref. [11], followed by Refs. [12,13,14,15,17], also performed homology modeling using *MODELLER* and provided a structural framework used in this work.

**Highlight** **1** (Devising more realistic models). *As described in Section 2.1.2 and Section 2.1.3, the CLG Fibril model was improved into Bone Fiber, which is a better representation of the experimentally determined nanostructure of bone presented in Refs. [18,20,22].*

**Remark** **2** (D-period). *The CLG Fibril, which is derived from the 3HR2 PDB from Ref. [6], exhibits the D-period of the CLG structure along the direction of its principal axis (z) [8], Figure 1. This means that at least one gap and one overlap zone are present in the CLG Fibril’s UC, and consequently in the CLG Fiber and Bone Fiber models described next.*

#### 2.1.2. CLG Fiber

     As previously mentioned, the deposition of HA in the IFV yields the mCLGf. However, as shown in Refs. [18,19,20,22,23], it is important to emphasize that most of the HA is found not in the IFV, but between and around fibrils, in the EFV. The results of Refs. [18,19] corroborate estimations exhibited in Ref. [21]; for cortical bone, about 70–80% of the HA content is situated in the EFV in a plate-like shape.

To the best of our knowledge, there are, as of yet, no available studies reporting MD simulations of the bone structure while taking into consideration the HA content in the EFV. There are probably two main reasons for this:

(a) The 3HR2 structure (and others derived from it, such as the presented CLG Fibril model) does not directly allow the deposition of HA in the EFV, but only within the fibril. That is because the UC defined by [6] possesses CLG covalent bonds that require PBCs in all directions. There is no room left for molecules in the EFV, and if the UC is expanded along the x and y directions to make space for such molecules, these would block the path of the CLG covalent bonds that require PBCs in the radial directions (x and y);
(b) Including HA in the EFV means devising a very large system (much larger than the UC of the 3HR2 structure), which implies computationally more expensive simulations.

Refs. [12,14,15], for example, do include HA in their models, but only in the IFV; i.e., the mCLGf is modeled by inserting HA crystals to the UC of a homology model similar to the CLG Fibril described here.

**Open Issue 1** (Coarse-Grained Models). *An alternative to simulate the CLG fiber structure without requiring a prohibitively large number of atoms is to use coarse-grained models where an entire group (typically from three up to five atoms) is treated as a single interacting entity [7,8,43,44]. Reference [43] presents a coarse-grained model of CLG molecules (including the non-standard amino acid HYP) using an extended version of the MARTINI force field [45]. Coarse-grained models combining CLG, $H_{2}O$, and HA are still an open field of research.*

The first step to create a model that resembles the structure of the fibers present in bone is to extract from the CLG Fibril a structure that requires no PBCs along the x and y direction, labeled here as CLG NanoFiber, as shown in Figure 2. After that, the desired model is obtained by replicating the latter along the x and y directions and inserting it into an EFV, i.e., a volume large enough to contain extra-fibrillar $H_{2}O$ and HA, the boundaries of which are subjected to PBCs. In Appendix B, a description is given on how to devise this structure, labeled as CLG Fiber.

#### 2.1.3. Bone Fiber

     When $H_{2}O$ and HA molecules are added to the CLG Fiber model described in Section 2.1.2, which contains an EFV, the newly devised model is labeled Bone Fiber.

**Remark** **3** (CLG Fiber vs. Bone Fiber models). *A bundle of axially aligned CLGfs and mCLGfs surrounded by $H_{2}O$ and HA characterizes bone at the nanoscale. The literature usually refers to this bundle as a CLG fiber. Throughout this paper, to avoid misunderstanding and to facilitate the understanding of the modeling process, a CLG Fiber model refers to a bundle of CLGfs (without $H_{2}O$ and HA). A Bone Fiber model refers to a bundle of hydrated mCLGf surrounded by $H_{2}O$ and HA in the EFV. Thus, here a Bone Fiber model refers to the CLG Fiber model plus $H_{2}O$ and HA, i.e., a composite material composed of fibers (CLG, $H_{2}O$, and HA) and a matrix ($H_{2}O$ and HA).*

The mechanical properties of bones at the nanoscale are affected by the relative fractions of their constituents. All models presented here consider bone to be constituted of CLG, HA, and H${}_{2}$O only; i.e., they consider the whole organic phase to be CLG and the whole inorganic phase to be HA. Four models were devised, each with a specific percentage of mass based on the reference values in Section 1.2 [22,30,31,32], as shown in Table 1.

**Open Issue 2** (Even More Realistic Bone Models). *The presented models consider bone to be constituted of CLG, HA, and $H_{2}O$ only. However, about 10% of the bone organic phase exhibits an association of other collagen types (III and VI), and non-collagenous proteins (NCPs) [2]. Furthermore, parts of the mineral phase may exhibit some deficiencies in hydroxyl, and also substitutes for hydroxyl which leads to the formation of other types of minerals, not only what is commonly labeled hydroxyapatite [4,46]. Both these variations may not represent a large fraction of the total organic and mineral phase and they are not simple to model, but they might affect the computed mechanical properties. Recently, Ref. [47] reported the implications of extra-fibrillar NCPs on the bone mechanical properties.*

Packmol, a package distributed as free software for building initial configurations for MD simulations [48], was used to add HA and $H_{2}O$ molecules to the CLG Fiber model, obeying the percentages of mass shown in Table 1. Note that the devised Bone Fiber models were labeled based on their HA concentration. Details on how to devise these models, and on how to compute the number of molecules of each constituent to be added to the simulation box are provided in Appendix C and Appendix D.

In all devised models, the total number of HA molecules was added to the simulation box such that 80% belong to the EFV, and only 20% to the IFV, as Refs. [18,19,20,21,22,23] point out. Packmol allows the creation of different geometries, including parallelepiped, sphere, cylinder, and other geometric shapes within which the new molecules will be inserted. The IFV was defined as a parallelepiped region within the larger simulation box, where CLG fibrils are mostly inside.

**Figure 5:**
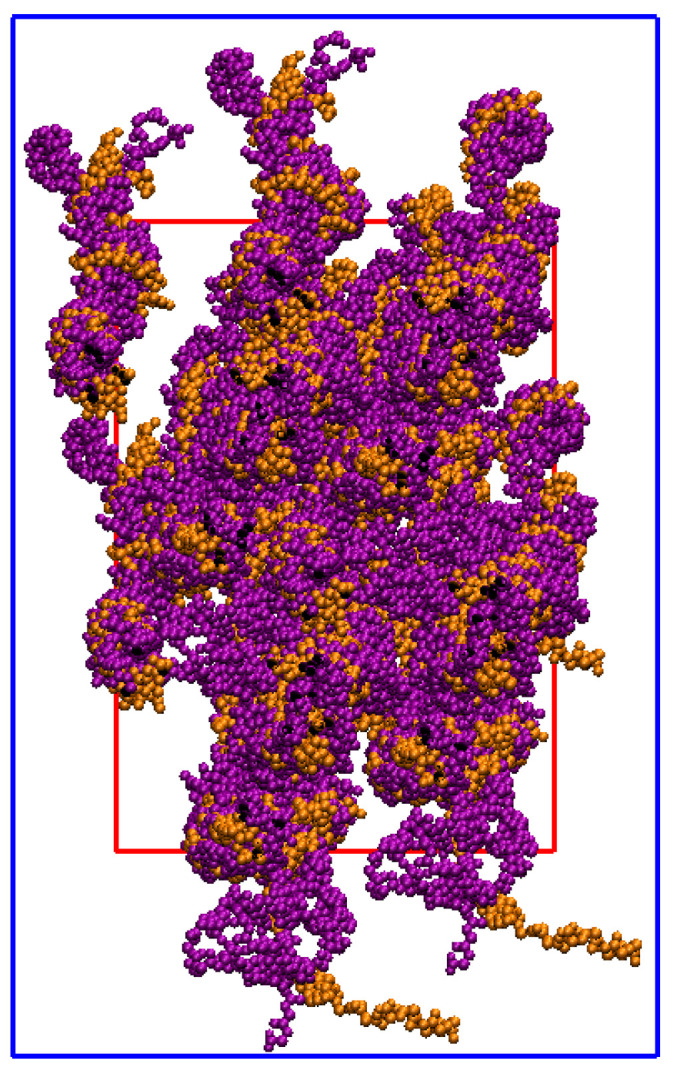
Bone Fiber view of the xy-plane (VMD). The simulation box (blue) defines the external boundary of the EFV. The IFV box (red) defines the external boundary of the IFV and the internal boundary of the EFV. Only CLG backbone molecules are shown.

**Figure 6:**
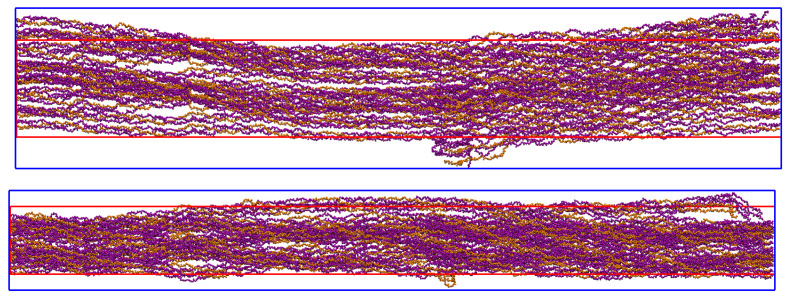
Bone Fiber view (VMD) of yz-plane (**top**) and xz-plane (**bottom**). The simulation box (blue) defines the external boundary of the EFV. The IFV box (red) defines the external boundary of the IFV and the internal boundary of the EFV. Only CLG backbone molecules are shown.

Figure 5 and Figure 6 show the boxes that define the IFV and EFV.

The devised IFV displays the x, y, and z dimensions 60×86×678 Å, and the simulation box dimensions 88×142×679 Å. This indicates that the length of the simulated fibers is 679 Å.

Note that 20% of the HA molecules were added into the IFV box, and the remaining 80% were outside the IFV, but inside the simulation box. The EFV is defined as the volume of the simulation box subtracted from the volume of the IFV box.

**Open Issue 3** (EFV vs. IFV). *By visually identifying the volume mostly occupied by the CLG fibrils, two different boxes were created that define the IFV and EFV. However, there may be more accurate ways to define the IFV and EFV for MD simulations. This paper presents a realistic model of a bone fiber (not fibril), i.e., the first model to reproduce fibrils and to insert HA molecules both in the IFV and in the EFV. However, modeling both the IFV and EFV can be considered an open issue.*

All the devised models display a salt concentration of 0.16 mol/L. This was assured by adding a total of 132 chloride ions and 0 sodium ions to the models (these 132 atoms are already included in the number of atoms shown in Table 1).

**Figure 7:**
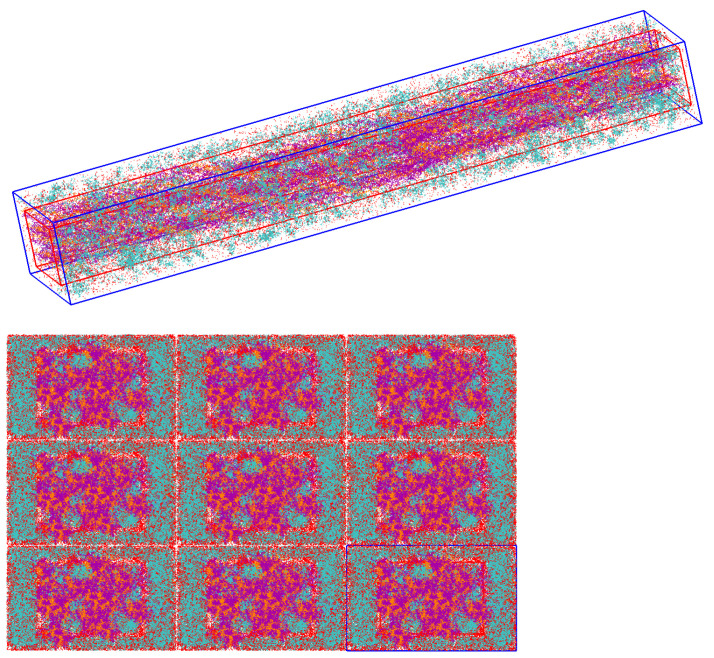
Simulation box of a Bone Fiber model, and a view of a 3-by-3 periodic replication of its xy-cross-section (VMD). HA, H${}_{2}$O and CLG molecules are shown in cyan, red, and purple (alpha-1 chains) and orange colors (alpha-2 chains), respectively.

**Figure 8:**
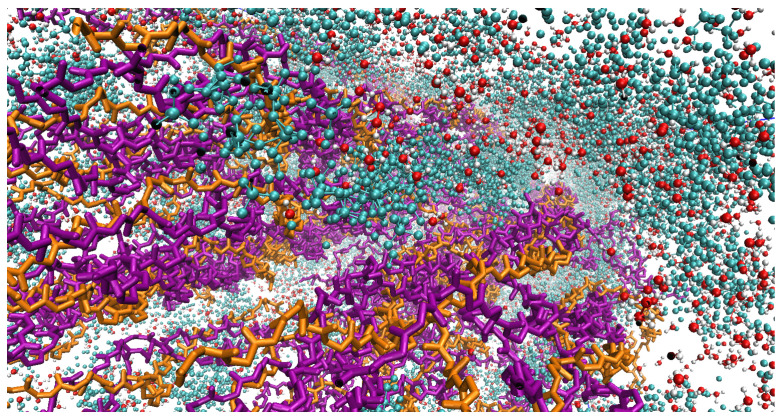
Zoomed view of a bone fiber in VMD. HA, H${}_{2}$O and CLG molecules are shown in cyan, red, and purple (alpha-1 chains) and orange colors (alpha-2 chains), respectively.

Figure 7 and Figure 8 show a devised Bone Fiber model, i.e., mCLGfs immersed in water and surrounded by HA inside the EFV. HA molecules from the INTERFACE force field (IFF) [49] database were used. Appendix E provides more detail about the used HA PDB file.

Notice that Ref. [42], Figure 1c, and Ref. [50], Figure 1d also extracted a CLG NanoFiber from the 3HR2 PDB structure provided by Ref. [6]; see Appendix B, Step 2. However, they do not further develop the model into a bone fiber structure, i.e., into a model such as the presented CLG Fiber or Bone Fiber.

### 2.2. Force Fields

Force fields (FFs) can significantly affect MD simulation results. It is thus paramount to select FFs that are appropriate for the specific goal of the simulation [51].

CHARMM36m [52,53,54,55], a well-known and tested FF especially developed for proteins, lipids, and carbohydrates, was selected. The files:

- *top_all36_prot.rtf, par_all36m_prot.prm* for proteins;
- *toppar_all36_prot_modify_res.rtf* for modified residues, i.e., HYP;
- *toppar_water_ions.prm* for water and ions;
  were used for the simulations described in this article, and included in the MODELLER 9.25 library during the homology modeling process, as described previously in Section 2.1.1 and Appendix A.

It is important to mention that the files *par_all36_lipid.prm, par_all36_carb.prm, par_all36_*

*na.prm, par_all36_cgenff.prm, and par_HA.prm*, though not containing parameters for the atoms of the presented models, were also loaded in the NAMD configuration files, since CHARMM files contain NBFIX, and CHARMM commands specifically written for the CHARMM program, not for NAMD. Reading all these files avoids errors in NAMD.

For the HA species, parameters from the IFF [49], which operates as an extension of CHARMM, were used. The parameters of the triclinic UC for HA are: *a* = 9.417 Å; *b* = 9.417 Å; *c* = 6.875 Å; α = 90${}^{{}^{\circ}}$; β = 90${}^{{}^{\circ}}$; γ = 120${}^{{}^{\circ}}$. See Appendix E for further details.

### 2.3. Minimization and Equilibration

Once devised, the Bone Fiber structure went through minimization steps and equilibration runs in NAMD before starting the production run; see Definition 4.

**Definition** **4** (Production Run). *There is a subtle difference between equilibration or thermalization and production runs. Both basically consist in running MD simulations (solving Newton’s Second Law for each atom in the system). However, data is only collected in the production run, since the computed properties should correspond to a system in thermodynamic equilibrium.*

MD simulations consist in solving Newton’s 2nd Law of Motion at a material molecular scale whose spatial domain contains *a* atoms interacting with up to *n* neighbor atoms:

(1) $$\;m_{a}\frac{d^{2}r_{a}\left(t\right)}{dt^{2}}=\sum_{n_{1}=1}^{n}f_{2}\left(r_{a}\left(t\right),r_{n_{1}}\left(t\right)\right)+\cdots +\sum_{n_{1}=1}^{n}\sum_{\begin{array}{c} n_{2}=1\\ n_{2}\neq n_{1} \end{array}}^{n}\cdots \;\sum_{\begin{array}{c} n_{k}=1\\ n_{k}\neq n_{1},n_{2}... \end{array}}^{n}\;f_{n}\left(r_{a}\left(t\right),r_{n_{1}}\left(t\right),\cdots ,r_{n_{k}}\left(t\right)\right)$$

where, for each a-th atom: $m_{a}$ is the mass, $r_{a}$ is the position vector, and $f_{2}$ is a force vector function that describes pairwise atomic interactions; similarly, $f_{n}$ describes n-atom interactions. Each $f_{n}$ is the spatial-derivative of a potential energy function that accounts for up to n-body and quantum interactions. The total energy of the a-th atom is a function of an a-th atom’s position $r_{a}\left(t\right)$ and its n neighbors’ positions $r_{1}\left(t\right),\ldots ,r_{n}\left(t\right)\in R^{3}$.

Details of the minimization and equilibration performed in NAMD and their parameters are shown in Table 2. Further information on the parameters can be found in the NAMD user guide.

**Figure 9:**
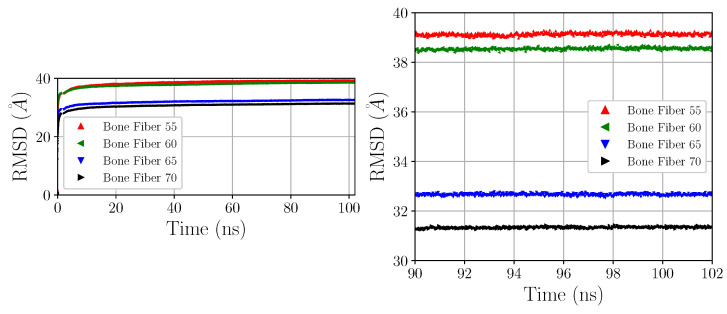
RMSD of Bone Fiber models (with respect to devised models, frame 0).

Structural convergence was ensured by analysis of the root mean squared deviation (RMSD), a numerical measure of the difference between two structures, of the CA atoms. The slope of the RMSD with respect to time approached zero short before 100 ns of equilibration. Figure 9 displays the computed RMSD for the devised Bone Fiber model.

**Remark** **4** (Volume Contraction). *During equilibration, a volume contraction varying from 30 to 50% with respect to the devised models was noticed. The volume contraction reflects a structural relaxation that is made possible by simulating in the NPT ensemble, which keeps the number of particles, pressure, and temperature constant, allowing the volume to adapt. Moreover, differently from other works that fully solvated the CLG molecule in water, here, a pre-defined number of water molecules was set to guarantee the relative composition of the nanomaterial, as shown in Table 1.*

LAMMPS, an open-source code with a focus on materials modeling and science [56,57,58,59,60,61,62,63], is among the most suitable code to study elastic properties of molecular models, including soft matter such as polymers and biomolecules such as CLG. As described in Section 2.4, LAMMPS was used for the computation of the Young’s Modulus of the devised models. A short additional equilibration using LAMMPS was also needed prior to the calculation of the elastic properties. The structurally stable (or simply relaxed) Bone Fiber structures were converted to LAMMPS using *charmm2lammps.pl* from LAMMPS tool. The LAMMPS equilibration consisted of: 1 ns equilibration with time step 1 fs and neighbor skin 1.0, followed by an additional 5 ns equilibration with a time step of 2 fs, as indicated in Table 3. Further information on the parameters can be found in the LAMMPS user guide.

PBCs were applied in all directions and during all steps.

### 2.4. Elastic Properties

     Assessing elastic properties using MD simulations is sometimes difficult [64,65], especially for biological systems, including proteins such as CLG. Nevertheless, a series of studies have been reported describing different techniques to address this problem [14,15,16,17]. Here, LAMMPS scripts were written which deform the simulation box in a manner that mimics uniaxial tensile tests.

     A uniaxial deformation along the z-axis was imposed by gradually increasing the z-length value of the simulation box, i.e., of the domain. Taking advantage of the continuum mechanics and strength of materials, the engineering strain along the z direction can be defined as:

(2) $$\varepsilon_{zz}\left(t\right)=\frac{L_{z}\left(t\right)-L_{z}\left(t_{0}\right)}{L_{z}\left(t_{0}\right)}=\frac{L_{z}\left(t\right)-L_{z0}}{L_{z0}}$$

where $L_{z}\left(t_{0}\right)=L_{z0}$ is the initial (t=0 s) length of the box along the z direction, and $L_{z}\left(t\right)$ is the length of the box along the z direction at time t. The engineering strain rate can be written as:

(3) $$\begin{array}{cc} {\dot{\varepsilon }}_{zz}\left(t\right) & =\frac{d\varepsilon_{zz}\left(t\right)}{dt}=\frac{d}{dt}\left(\frac{L_{z}\left(t\right)-L_{z0}}{L_{z0}}\right)=\frac{dL_{z}}{dt}\frac{1}{L_{z0}}=\frac{v_{z}\left(t\right)}{L_{z0}} \end{array}$$

where $v_{z}\left(t\right)$ is the velocity with which the box z length changes over time. The LAMMPS *fix deform* command deforms the box by extending the box length $L_{z}$, at each time step t, following:

(4) $$\begin{array}{c} L_{z}\left(t\right)=L_{z0}\left({\dot{\varepsilon }}_{zz}\left(t\right)\cdot t+1\right)=v_{z}\left(t\right)\cdot t+L_{z0}. \end{array}$$

LAMMPS allows the user to decide whether to input the strain rate ${\dot{\varepsilon }}_{zz}\left(t\right)$ or velocity $v_{z}\left(t\right)$. Here, a constant strain rate of $10^{-5}\left[1/fs\right]$ was set. Since a box extension of 30%, $L\left(t_{final}\right)=1,3L_{0}=L_{0}\left(10^{-5}\cdot t+1\right)$, is more than sufficient to assess the elastic properties of such a system through MD simulations, a total deformation run time of 30 ps was used. Table 4 shows the main parameters used for the tensile test simulations.

**Figure 10:**
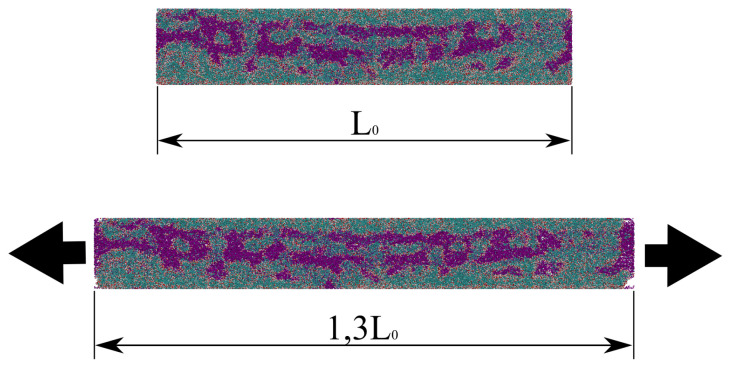
Bone Fiber 55 UC before (**top**) and after (**bottom**) the tensile test; snapshots from OVITO [66]. The arrows indicate the stretching directions.

PBCs were applied in all directions and during all steps of the production run. While the box was deformed along the z direction, an NPT ensemble was used for the x and y ones. Figure 10 shows the UC of the Bone Fiber 55 model before and after being uniaxially deformed by 30%.

Assuming bone as a Cauchy-Linear-Elastic (CLE) material [2] complying with Hooke’s Law, a tensile test allows the estimation of the Young’s Modulus *E* through the following stress-strain relationship:

(5) $$\sigma_{zz}=E\varepsilon_{zz}$$

The LAMMPS default *compute pressure* command computes the elements of the symmetric pressure tensor at the molecular scale by adding components of the kinetic energy tensor and of the virial tensor:

(6) $$P_{ij}=\frac{\sum_{k=1}^{N}m_{k}v_{ki}v_{kj}}{V}+\frac{\sum_{k=1}^{N^{\prime }}r_{ki}f_{kj}}{V}$$

where N is the number of atoms ($N^{\prime }$ includes atoms from neighboring sub-domains, labeled ghost atoms), $m_{k}$ is the mass of the k-th atom, $v_{ki}$ the i-th component of the velocity of the k-th atom, $r_{ki}$ the i-th component of the position of the k-th atom, and $f_{ki}$ the *i*-th component of the resultant force applied on the k-th atom. Here, pressure can be interpreted as stress; i.e., $P_{ij}=\sigma_{ij}$.

## 3. Results and Discussion

**Figure 11:**
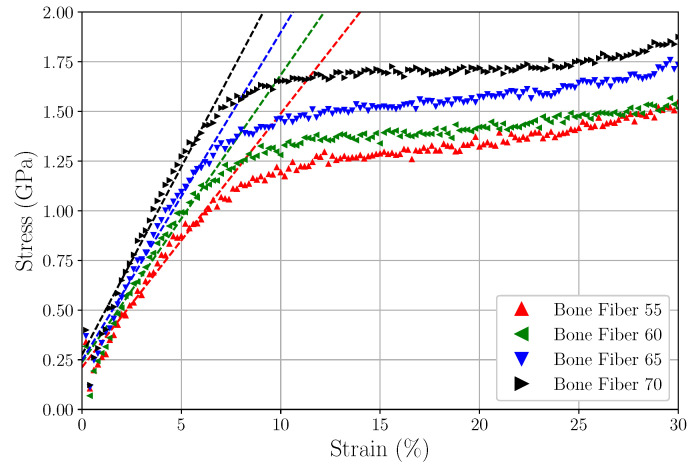
Stress–strain curves computed for the devised models, and their respective linear regression.

During the MD tensile tests simulations, stress and strain were frequently outputted and later plotted to strain-stress curves. Figure 11 shows the stress–strain curves obtained from MD simulation using the LAMMPS *fix deform* command and the respective linear fitting of the elastic region.

A simple linear regression based on least squares using *scipy.optimize.curve_fit* [67] was used to compute the lines that fit the elastic region of the models (adopted as the region between 1 and 7% of strain), and consequently the estimatives of Young’s Modulus values, defined as the slope of the lines. Table 5 displays the estimated Young’s Modulus values for the devised Fiber models.

Here, bone was considered a CLE material complying with Hooke’s Law. No plastic, viscoelastic, or non-linear behavior was considered. The Young’s Modulus values shown in Table 5 were compared with those presented in the literature. A discussion on how they can be interpreted is provided below.

Ref. [4] compares Young’s Modulus values calculated for CLG at different length scales applying different methods. They presented Young’s Modulus values ranging between 0.35 and 12 GPa for their classification of the molecular scale, between 0.2 and 38 GPa for their classification of the microfibrillar/fibrillar scale, and between 0.03 and 1.57 GPa for their classification of fiber scale. The large range and difference between the presented Young’s Modulus values can be explained by the different applied methodologies (molecular dynamics, X-ray diffraction, atomic force microscope, and others).

Ref. [14] performed MD simulations to compute Young’s Modulus values for mCLGf models with different concentrations of HA and $H_{2}O$, obtaining values ranging from 0.2 to 1.9 GPa. Furthermore, Ref. [14] displays a compilation of Young’s Modulus values ranging from 0.2 to 2.8 GPa for mCLGfs computed using both experimental and computational methods. Reference [15] also displays a compilation of Young’s Modulus values, this time compressive, ranging from 0.03 to 22.11 (13.87 + 8.24) GPa for mCLGfs computed using both experimental and computational methods.

Refs. [68,69] devised continuum multiscale models and obtained homogenized stiffness tensors for nanoscale models (see [68] Appendix B), which also agrees with the presented literature, and thus with our results.

As shown above and also discussed by ref. [70,71], there is no standard value for the Young’s Modulus of CLGf, mCLGf, and CLG fibers. The literature presents values that differ more than 100% from each other and also do not precisely classify the applied length scale. What one reference classifies as microfibril, is sometimes classified as fibril by another reference; see Remark 1.

As discussed in Appendix B, Open Issue A1, the model labeled Bone Fiber possesses too few CLG molecules when compared to a real CLG fiber. However, it is the most realistic model that has, to our knowledge, been devised to date. It displays 20 CLG single molecules (tropocollagens), in the overlap region, 16 in the gap region, and includes HA molecules both in the IFV and in the EFV. A rigorous classification places the devised Bone Fiber models somewhere between mCLGfs and CLG fibers, so the computed Young’s Modulus should lay in the range between these two; i.e., any value between 0.03 to ∼20 GPa can be considered reasonable.

Nevertheless, the presented approach allows the modeling of larger, and even more realistic bone nanoscale fiber model. Unfortunately, the almost prohibitive computational cost of these models precludes its large use, since this would require millions, and even billions, of atoms.

## 4. Conclusions

Although earlier experiments showed that fibers in bone exhibit most of their HA in the EFV [18,20], no molecular model regarding this feature has been presented in the literature. We present for the first time all-atom bone models that include HA both in the IFV and in the EFV, i.e., more elaborate bone nanoscale models from a biological point of view. Our purpose is to provide a detailed prescription on how to devise such models with different fractions of their basic constituents. Thus, we provide all used scripts as well as the PDB and PSF files of the equilibrated structures (∼100 ns) in the Supplementary Materials.

We performed simple tensile tests using LAMMPS in order to assess the Young’s Modulus values of the devised models. Our results are in good agreement with the literature, although the data reported by different groups for bone-like nanostructures fall over a broad range of values. Future computational and experimental studies could provide additional validation.

By including HA in the EFV, the present Bone Fiber models take into account an important element of the biology and chemistry of fibers in bones, and can be easily modified to model larger and even more human-like bone fibers. The models unfold a new alternative to study the nanoscale mechanics of bones, and together with the information provided in this work, can be used as the foundation of future studies regarding the modeling and mechanical properties of bone at the nanoscale.

## Figures and Tables

**Table 1:** Devised Bone Fiber models, the mass percentages of the bone constituents, and their total number of atoms.

Model Name	HA %	CLG %	H${}_{2}$O %	Number of Atoms
Bone Fiber 55	55	35	10	299136
Bone Fiber 60	60	30	10	331797
Bone Fiber 65	65	25	10	377486
Bone Fiber 70	70	20	10	446018

**Table 2:** Parameters of MD simulation for minimization and equilibration in NAMD.

Parameter Name	Parameter Value
Minimization Algorithm	Conjugate Gradient
Equilibration Time	100 ns
Equilibration Time Step	2.0 fs
Equilibration Ensemble	NPT
Cutoff	12.0 Å
Switch distance	10.0 Å
Pair list distance	14.0 Å
Particle-Mesh Ewald Sum Grid Spacing	1.0 Å
Temperature Control Algorithm	Langevin Dynamics
Constant Temperature	310 K
Pressure Control Algorithm	Nosé–Hoover Langevin Piston
Constant Pressure	1.01325 bar

**Table 3:** Parameters of MD simulation for minimization and equilibration in LAMMPS.

Parameter Name	Parameter Value
Equilibration Time	5 ns
Equilibration Time Step	2 fs
Equilibration Ensemble	NPT
Inner Cutoff	12.0 Å
Outer Cutoff	14 Å
Neighbor Skin	2.0 Å
Particle-Particle Particle-Mesh Solver Desired Relative Error in Forces	1 × 10${}^{-6}$
Temperature Control Algorithm	Langevin Dynamics
Constant Temperature	310 K
Pressure Control Algorithm	Nosé–Hoover
Constant Pressure	1.0 atm

**Table 4:** Parameters of MD simulation for tensile tests in LAMMPS.

Parameter Name	Parameter Value
Deformation Time	30 ps
Deformation Time Step	2 fs
Deformation Direction	z
Strain Rate	1 × 10${}^{-5}$ $\frac{1}{fs}$
Equilibration Ensemble	NPT
Inner Cutoff	12.0 Å
Outer Cutoff	14 Å
Neighbor Skin	2.0 Å
Particle-Particle Particle-Mesh Solver Desired Relative Error in Forces	1 × 10${}^{-6}$
Temperature Control Algorithm	Langevin Dynamics
Constant Temperature	310 K
Pressure Control Algorithm (in x and y)	Nosé–Hoover
Constant Pressure	1.0 atm

**Table 5:** Computed Young’s Modulus values for devised Bone Fiber models.

Model Name	Young’s Modulus [GPa]
Bone Fiber 55	12.77
Bone Fiber 60	14.45
Bone Fiber 65	16.52
Bone Fiber 70	18.90

**Figure A1:**
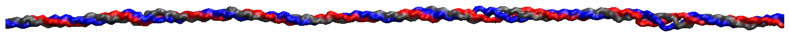
Model of human triple-helix CLG structure devised by homology modeling using MODELLER and shown in VMD with drawing and coloring methods Quicksurf and Chain, respectively.

**Figure A2:**
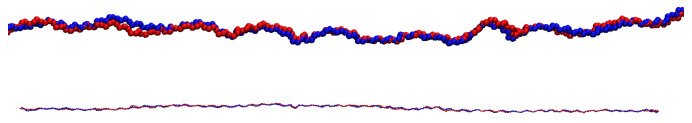
Chains A of the lowest DOPE model built with *allhmodel* (blue) and 3HR2 (red) are shown in VMD as VDW. Zoomed (**top**) and distant (**bottom**) views.

**Figure A3:**
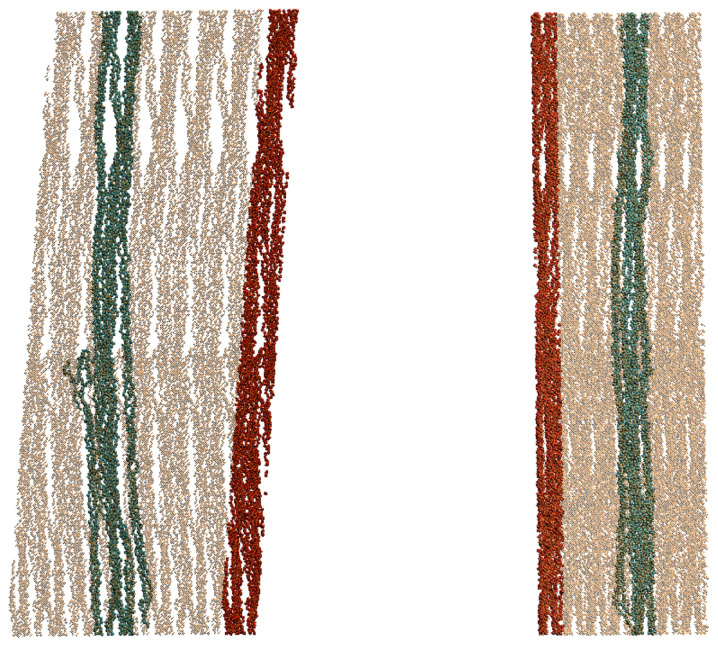
Extraction of CLG NanoFiber (blue) from the seven-by-seven periodic replication (orange transparent) of the CLG Fibril (red). View of xz-plane (**left**) and yz-plane (**right**) in VMD.

**Figure A4:**
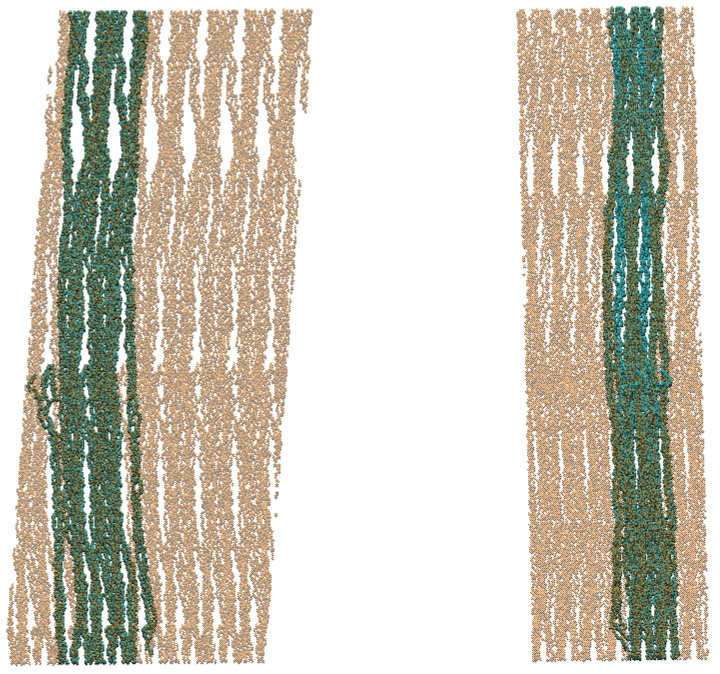
CLG Fiber (blue): a two-by-two replication of CLG NanoFiber, seen among the seven-by-seven periodic replication of the CLG Fibril’s UC (orange transparent). View of xz-plane (**left**) and yz-plane (**right**) in VMD.

**Figure A5:**
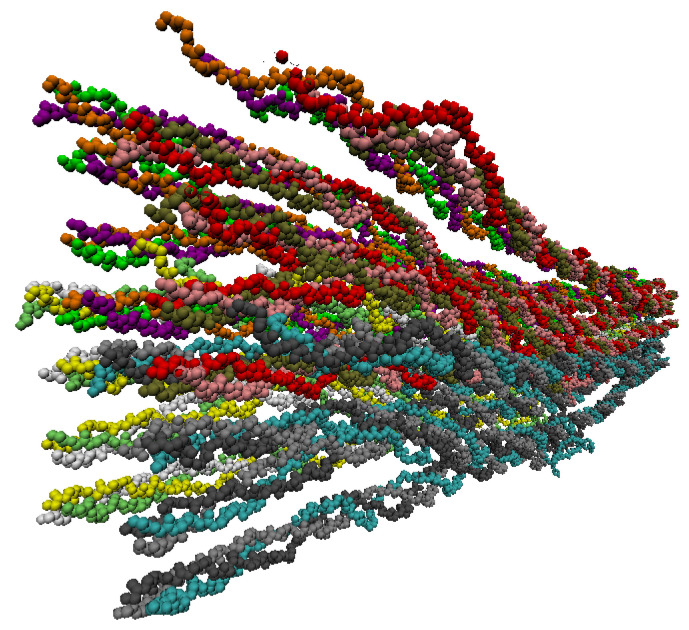
Devised CLG Fiber shown in VMD. The CLG chains (three chains each in one color) of a previous specific CLG NanoFiber are all represented five times (in the overlap zone) by the same three colors, e.g., five CLG molecules with their three chains in gray, cyan, and black. The different replications of the previous CLG NanoFiber can be identified by the colors cyan, red, yellow, and orange.

## Data Availability

The data presented in this study are available in the Supplementary Materials.

## Supplementary Materials

The following supporting information can be downloaded at: https://www.mdpi.com/article/10.3390/ma15062274/s1, (**0-COL1_ Modeller**.)—files and scripts used to perform homology modeling using MODELLER 9.25; (**1-CLG_  Fibril**.)—files and scripts used to devise the CLG Fibril model; (**2-CLG_  Fiber**.)—files and scripts used to devise the CLG NanoFiber and CLG Fiber models; (**3-Bone_ Fiber**.)—files and scripts used to devise CLG Bone Fiber models and equilibrate it. In the latter, we also provide PDB and PSF files of the equilibrated Bone Fiber models.

## Abbreviations

   The following abbreviations are used in this manuscript:

CA	Alpha Carbon
CHARMM	Chemistry at HARvard Macromolecular Mechanics
CLE	Cauchy-Linear-Elastic
CLG	CoLlaGen
CLGf	CoLlaGen fibril
mCLGf	mineralized CoLlaGen fibril
EFV	Extra-Fibrillar Volume
FF	Force Field
GLY	GLYcine
HA	HydroxyApatite
HPC	High Performance Computing
HYP	HYdroxyProlyne
IFF	Interface Force Field
IFV	Intra-Fibrillar Volume
LAMMPS	Large-scale Atomic/Molecular Massively Parallel Simulator
NAMD	NAnoscale Molecular Dynamics
NCP	Non-Collagenous Protein
OVITO	Open VIsualization TOol
PBC	Periodic Boundary Condition
PDB	Protein Data Bank (also a file format)
PRO	PROline
PSF	Protein Structure File (a file format)
RMSD	Root Mean Squared Deviation
UC	Unit Cell
UniProt	Universal Protein resource
VMD	Visual Molecular Dynamics

## Appendix A. Building Homology Models of Human Type-I Collagen Using MODELLER 9.25

There are five main steps:

1. Select *target* sequences and properly prepare the data;
2. Select *template* structures and properly prepare the data;
3. Align *target* and *template* sequences;
4. Build models;
5. Check the models.

All used files and scripts are available in the Supplementary Materials.

### Appendix A.1. Selecting Target Sequence and Preparing Its Data

The COL1A1_human (P02452) and COL1A2_human (P08123) amino acid sequences were selected as targets. As mentioned before, they can be found on the UniProt website. Since only the CLG chains need to be modeled, i.e., without signal peptide and propeptide, only the residues in positions 162 to 1218 (feature identifier PRO_0000005720) are needed for COL1A1, and residues 80 to 1119 (feature identifier PRO_0000005805) for COL1A2. Furthermore, these sequences also contain modified residues, i.e., non-standard amino acids such as HYP. Although UniProt indicates the position of each non-standard amino acid, it exports the sequence with the respective unmodified residue in the FASTA format. Specific PROs located at the third position of the tripeptide repeating unit GLY-X-Y were manually substituted with HYPs. This was done by replacing the specific letter P with the letter O.

Finally, the modified human CLG sequence was converted to the PIR format (MODELLER’S preferred format for comparative modeling) for later alignment with the rat sequence.

### Appendix A.2. Selecting Template Structure and Preparing Its Data

The rat CLG structure (3HR2) was selected as the template. As mentioned before, it can be found in the PDB. The 3HR2 structure contains two non-standard amino acids: HYP (4-Hydroxyproline) and LYZ (5-Hydroxylysine). To include them in the final model, their topology and force field parameters files must be included in MODELLER’s library. Since LYZ appears only a few times in the structure and is not paramount to the fibrillar structure, the 3HR2.pdb file was manually edited by substituting all LYZ by LYS (Lysine), a standard amino acid. HYP, on the other hand, was not removed because it is very abundant in the CLG and plays an important role in the formation of the fibrillar structure. However, MODELLER does not automatically identify the non-standard amino acids (HETATM) when reading the sequence of a PDB file. It is possible only by appropriately editing MODELLER’s scripts, and the library file *restyp.lib*. See the available README.txt files in the Supplementary Materials for details.

A MODELLER script was used to extract the sequence in the PIR format from the template structure. This sequence was then added to an input alignment file (.ali) containing the target sequence as well.

### Appendix A.3. Aligning Target and Template Sequences

In MODELLER 9.25, there are two types of alignment (*.ali) files. There is an *input alignment file* containing the non-aligned sequences of both target and template and an *output alignment file* containing the aligned sequences of both target and template. An alignment script performs the alignment of the input alignment file into an output alignment file.

In the input alignment file containing the non-aligned target and template sequences, several “-” were manually added to the template sequences of the rat CLG so that they could exhibit the same lengths as the target human sequences (1057–1040–1057 for chains A, B, and C, respectively). This input alignment file was used as input for the alignment of the sequences, which is described next.

**Remark** **A1** (Human sequence length vs. Rat sequence length). *To devise models with the length of the target* *Homo sapiens* *(Human) sequence, "-" was manually included in the template sequences. To devise models with the length of the template* *Rattus Norvegicus* *(Brown Rat) sequence, a few residues from the target sequences were manually deleted. The latter were discarded since the goal was to devise models as close to the human type-I collagen as possible. We believe that models with the original human sequence length are a better representation of the real human collagen.*

Further details can be found in the scripts available in the Supplementary Materials.

### Appendix A.4. Building Models

Output align files become the input files for scripts that build homology models. Twenty models were built, ten using the *automodel* function and ten using the *allhmodel* function. It takes much longer to build models using *allhmodel* (it includes H atoms); however, since the used HYP topology and force field parameters (CHARMM36m) include H atoms, models with *allhmodel* were also built. Figure A1 shows the best model built with the *allhmodel* function. A comparison between the models built with each function and how the best model was chosen is described in the next step.

Force field parameters and topology files for HYP were added by editing MODELLER’s library files *par.lib*, *top_heav.lib/top_allh.lib*, *radii.lib*, *radii14.lib*, and *solv.lib*. The same CHARMM36m parameters and topology for HYP used here were later used for the MD simulations.

### Appendix A.5. Checking the Models

MODELLER provides some *assessment functions* so the user can assess the quality of the model. GA341 and DOPE are two examples of them. GA341 is recommended for single-chain proteins and a GA341 score below 0.7 indicates a “bad” model. DOPE is the most reliable at separating native-like models from decoys. DOPE score is calculated based on energy, meaning that smaller values are better.

However, the best model among the models built by MODELLER does not necessarily mean a “good” model. It is also important to compute the RMSD between the built model and template structure and to visualize and compare both using external software. Figure A2 shows chains A of both the built model and the 3HR2 template structure.

The RMSD between the CA atoms of the template structure, and the models built with both *automodel* and *allhmodel* were computed chain-by-chain. For the lowest DOPE model built using *automodel*, an average RMSD value of 5.5 Å was obtained. For the lowest DOPE model built using *allhmodel*, on the other hand, an average RMSD value of 4.5 Å. Hence, the lowest DOPE model built using *allhmodel* was selected. The likely reason being that the CHARMM36m force field topology for HYP is added to the MODELLER’s library. It includes hydrogen atoms and, therefore, MODELLER builds better models when this information is added. To create models using *automodel*, i.e., with no hydrogen atoms, the hydrogen lines from the topology file for HYP had to be manually commented out.

## Appendix B. CLG Fiber

The CLG Fiber model consists of a bundle of CLG Fibril models surrounded by $H_{2}O$ and HA along the x and y directions. However, simply doing this is not possible. The atoms of the CLG Fibril covalently bonded through the PBCs of the UC would be far apart from each other. Their covalent bonds would clash with $H_{2}O$ and HA. This issue can be solved by exploiting the *minimum-image convention*. Below, a three-step description is given of how the CLG Fiber was devised starting from the CLG Fibril:

1. Replicate the CLG Fibril in the x and y directions.

To use the minimum-image convention, images of the UC must be generated. Thus, the CLG Fibril’s UC was replicated along the x and y directions using the script *replicateCrystal.tcl* with a few subtle modifications. The original script belongs to the Bionanotechnology Tutorial available on the NAMD website [72,73] (http://www.ks.uiuc.edu/Research/namd/ accessed on 30 January 2022). The UC was replicated seven times along the x and y directions, thus creating a so-called supercell of 49 UC images that guarantees enough molecules to extract the desired structure. It can be seen from Figure 3C of Ref. [6] that a single CLG molecule goes through seven UCs along the x direction (represented there by the letter a). For example, trying to do the same with a three-by-three reproduction of the CLG Fibril did not yield a final model that requires no PBC along the x and y directions.

2. Extract the CLG NanoFiber, a structure that requires PBCs along the z direction only.

A Matlab script was written which, starting from the middle UC image (located at the center of the seven-by-seven UCs), selects the first atom of each chain and searches for the next nearest atom in the chain among its possible 49 images. The pseudocode shown in Algorithm A1 details the main core of this Matlab script [74].

Figure A3 shows the extracted structure in cyan color, labeled CLG NanoFiber, among the seven-by-seven replication of the CLG Fibril.

**Algorithm A1** Extracting CLG NanoFiber from super cell of 49 CLG Fibril replications
▷ % For desired number of central box (cb), i.e., a UC located in center of the 49 images % **for** $j\leftarrow 1:n_{cb}$ **do** ▷ % From the 2nd to the last atom of the cb % **for** $j\leftarrow 2:n_{atom_{cb}}$ **do** cb_index← converts local index of cb (j) to cb global index **if** cb_index-th atom belongs to same chain as (cb_index-1)-th atom **then** index_set(1,49)← set of atoms in the *j*-th position in each of the 49 images xy_set(49,2)← (x,y) coordinates of atoms of the set index_set xy_ref(1,2)← (x,y) coordinates of (cb_index-1)-th atom (reference atom) ▷ % compute distance between atoms of the set index_set and the reference atom % $r_{set}\leftarrow sqrt\left(\left(xy_{set}\left(:,1\right)-xy_{ref}\left(1,1\right)\right).^{2}+\left(xy_{set}\left(:,2\right)-xy_{ref}\left(1,2\right)\right).^{2}\right)$ ▷ % get minimum distance % $\left[min_{val},min_{index}\right]\leftarrow min\left(r_{set}\right)$ ▷ % if nearest atom not already in cb % **if** $index\_set(1,min_{index})\neq cb\_index$ **then** Replace atom of the cb by nearest atom (from other UC) ! **end if** **else** ▷ % do nothing % ▷ % cb_index-th atom is 1st atom of chain and reference for (cb_index+1)-th atom % **end if** **end for** **end for**

The CLG NanoFiber is a structure that requires PBCs along the z direction only. This means that, differently from the CLG Fibril, $H_{2}O$ and HA molecules can be added around the CLG molecules in the EFV without clashing CLG covalent bonds.

3. Replicate the CLG NanoFiber along the x and y directions to produce the CLG Fiber.

Finally, using the same modified *replicateCrystal.tcl* script, the CLG NanoFiber was replicated two times along the x and y directions by a distance apart equivalent to the CLG Fibril’s UC, mentioned in Section 2.1.1. The final goal is to devise a model similar to the fiber structure described in Refs. [2,18,20,22], which can be regarded as a bundle of mCLG fibrils immersed in an EFV filled with H${}_{2}$O and HA. The inclusion of H${}_{2}$O and HA is described in Section 2.1.3 and detailed in Appendix C.

**Remark** **A2** (Skipping Step 3). *An alternative way to devise the same CLG Fiber model, but without performing Step 3 described above, is to adapt Algorithm A1 to extract not only one central box, but four boxes in the central area of the 49 images, i.e., $n_{cb}=4$. This way it directly provides a two-by-two replication of the CLG NanoFiber model. This adaptation is straightforward and provided in the Supplementary Materials. However, Algorithm A1 was not carefully optimized to run fast and, for instance, if a five-by-five replication is desired, we recommend replicating, as Step 3 shows, an extracted single central box, $n_{cb}=1$, instead of using Algorithm A1 to extract 25 boxes.*

The devised CLG Fiber (see Figure A4 and Figure A5) displays 20 CLG molecules in the overlap zone and 16 in the gap zone, since the CLG Fibril (derived from the PDB 3HR2 structure) displays five and four, respectively.

**Open Issue A1** (Larger and more realistic Fibers).
*A real CLG fiber possesses far more than just 20 CLG molecules. However, too many CLG molecules imply a very large UC and an elevated number of $H_{2}O$ and HA molecules. This is computationally expensive and demands time-consuming simulations, even when taking advantage of high-performance computing (HPC). However, a much larger bundle of CLG molecules that better represents the fiber structure of CLG can be easily produced following the procedures (and files) provided in the Supplementary Materials. The final CLG Fiber could be a three-by-three, five-by-five, or even ten-by-ten (500 CLG molecules in the overlap zone and 400 in the gap zone) replication of the CLG NanoFiber. The computer performance of the MD simulations is the main limitation. Note that no replication along the z direction is required. The PBC along the z direction and the CLG Fibril, which derives from the 3HR2 PDB, guarantee the D-period of the CLG; i.e., at least one gap and one overlap zone are included in the simulation box (see Remark 2).*

## Appendix C. Bone Fiber

Using the CLG Fiber model described in Section 2.1.2 and Appendix B, the following steps were performed to devise the Bone Fiber model.

1. Aligning the principal axes of the model to the x, y, and z directions.

This was done following instructions provided on https://www.ks.uiuc.edu/Research/vmd/script_library/scripts/orient/ (accessed on 30 January 2022);

2. Translating the center of the model to the origin of the Cartesian system.

This step is not mandatory, but working with the center of the simulation box positioned at the center of the Cartesian system, i.e., $\left(x_{center},y_{center},z_{center}\right)=\left(0,0,0\right)$, is a common procedure in MD simulations that facilitates some future computation and analysis;

3. Adding H atoms.

Though, as described in Appendix A, a homology model containing H atoms was selected (built with the *allhmodel* function), the H atoms were removed, as described in Section 2.1.1. Here, H atoms are added to the model through a script for the VMD psfgen plugin to generate a PSF and a new PDB file.

**Remark** **A3** (Bad contacts).
*After adding H atoms to the aligned and translated CLG Fiber model, a few minimization and equilibration steps (MD simulation runs) were performed in NAMD to assure that the model runs stably, and is suitable for further modifications. Here, a few bad contacts were found in the model. After a careful search, it was found that:*

(1) *Bad contacts between residues LEU2470 and HYP2708 appeared earlier in the CLG Fibril model after wrapping all atoms into the CLG Fibril’s UC, at Section 2.1.1, Step 3. A possible reason is the position of the side chains and H atoms determined by the homology modeling and the VMD psfgen plugin. By including the side chain and H atoms in a very dense UC, it is probable that the newly included atoms were positioned too close or even crossed other molecules. They do not necessarily avoid the crossing of different molecules (entangling);*
(2) *Bad contacts also appeared after replicating the CLG NanoFiber by distances equivalent to the CLG Fibril’s UC (or by directly extracting the CLG Fiber from the 49 images). After adding H atoms to the CLG Fiber model, a small number of molecules crossed the pentagonal structure of other molecules. The size of the CLG Fibril’s UC seems too short for the extracted NanoFiber structure accounting for the side chains and H atoms added by MODELLER and the VMD psfgen plugin, respectively.*

*All bad contacts were manually removed using the VMD shortcut 5.*

4. Adding $H_{2}O$ and HA using PACKMOL.

Packmol was used to add $H_{2}O$ and HA molecules to the aligned and translated CLG Fiber model so that predefined fractions of molecular mass are kept constant. A detailed description is given in Appendix C and Appendix D of how the number of water ($n_{H2O}$) and hydroxyapatite ($n_{HA}$) molecules to be added to the CLG Fiber model was calculated. Models with different mass percentages of CLG, HA, and $H_{2}O$ were devised, as shown in Table 1.

In all devised models the total number of HA molecules was added to the box such that 80% belongs to the EFV, and only 20% to the IFV. Figure 5 and Figure 6 visually differentiate the EFV and IFV.

Once the correct number of $H_{2}O$ and HA molecules were added to the simulation box, the newly devised model was labeled Bone Fiber. Again, the VMD psfgen plugin was used to generate a PSF and a new PDB file for the Bone Fiber model.

## Appendix D. Mass Fraction Calculation

Units: Volume (V) $\left[{Å}^{3}\right]$; Mass (m) [g]; Molar mass (mm) $\left[\frac{g}{mol}\right]$; Density (ρ) $\left[\frac{g}{ml}\right]$ = $\left[\frac{g}{cm^{3}}\right]$ = $\left[\frac{g}{10^{24}{Å}^{3}}\right]$.

Avogadro constant: $N_{A}=6.022\cdot 10^{23}\left[\frac{1}{mol}\right]$

Basic equations of micromechanics:

Mass conservation (m)

(A1) $$\begin{array}{c} m_{sys}=m_{CLG}+m_{HA}+m_{H2O},\\ \rho_{sys}\cdot V_{sys}=\rho_{CLG}\cdot V_{CLG}+\rho_{HA}\cdot V_{HA}+\rho_{H2O}\cdot V_{H2O},\\ \rho_{sys}=\rho_{CLG}\cdot {\mathrm{Vf}}_{CLG}+\rho_{HA}\cdot {\mathrm{Vf}}_{HA}+\rho_{H2O}\cdot {\mathrm{Vf}}_{H2O}. \end{array}$$

Volume conservation (V)

(A2) $$\begin{array}{c} V_{sys}=V_{CLG}+V_{HA}+V_{H2O},\\ \frac{m_{sys}}{\rho_{sys}}=\frac{m_{CLG}}{\rho_{CLG}}+\frac{m_{HA}}{\rho_{HA}}+\frac{m_{H2O}}{\rho_{H2O}},\\ \frac{1}{\rho_{sys}}=\frac{{\mathrm{Mf}}_{CLG}}{\rho_{CLG}}+\frac{{\mathrm{Mf}}_{HA}}{\rho_{HA}}+\frac{{\mathrm{Mf}}_{H2O}}{\rho_{H2O}}. \end{array}$$

Mass fraction (Mf)

(A3) $${\mathrm{Mf}}_{CLG}=\frac{m_{CLG}}{m_{sys}},\;{\mathrm{Mf}}_{HA}=\frac{m_{HA}}{m_{sys}},\;{\mathrm{Mf}}_{H2O}=\frac{m_{H2O}}{m_{sys}}.$$

(A4) $${\mathrm{Mf}}_{CLG}+{\mathrm{Mf}}_{HA}+{\mathrm{Mf}}_{H2O}=1.$$

Volume fraction (Vf)

(A5) $${\mathrm{Vf}}_{CLG}=\frac{V_{CLG}}{V_{sys}},\;{\mathrm{Vf}}_{HA}=\frac{V_{HA}}{V_{sys}},\;{\mathrm{Vf}}_{H2O}=\frac{V_{H2O}}{V_{sys}}.$$

(A6) $${\mathrm{Vf}}_{CLG}+{\mathrm{Vf}}_{HA}+{\mathrm{Vf}}_{H2O}=1.$$

The main goal is to add H${}_{2}$O and HA molecules to a molecular domain, i.e., the simulation box, previously with collagen molecules in vacuum only, so that predefined percentages of the constituent’s molecular masses are kept constant. For this, Packmol was used. Below a description is provided of how the number of water ($n_{H2O}$) and hydroxyapatite ($n_{HA}$) molecules was calculated. The inputs are predefined mass fractions of the constituents, their molar mass, and their initial density.

Desired mass fractions of bone constituents:

(A7) $${\mathrm{Mf}}_{CLG}\;{\mathrm{Mf}}_{HA}\;{\mathrm{Mf}}_{H2O}.$$

Values for the desired mass fraction were selected based on the literature [22,30,31,32], see Section 1.2. The selected values are shown in Table 1.

Density of constituents $\left[\frac{g}{ml}\right]$ = $\left[\frac{g}{cm^{3}}\right]$ = $\left[\frac{g}{10^{24}{Å}^{3}}\right]$

(A8) $$\rho_{CLG}=1.43,\;\rho_{HA}=3,\;\rho_{H2O}=1.$$

These density values were taken from Ref. [75], apud [76], and [77], apud [21,78].

Molar mass of constituents [g/mol]

(A9) $$\begin{array}{c} mm_{CLG}=1222226.78\left[\frac{g}{mol}\right],mm_{HAunit}=1004.62\left[\frac{g}{mol}\right],mm_{H2Ounit}=18.01\left[\frac{g}{mol}\right]. \end{array}$$

Values for $mm_{CLG}$ and $mm_{HAunit}$ were computed using the script solvate.tcl from Packmol’s utilities (http://leandro.iqm.unicamp.br/m3g/packmol/utilities.shtml, accessed on 30 January 2022).

Note that the mass of the constituents in [g] can be calculated by:

(A10) $$\begin{array}{c} m_{CLG}=\frac{mm_{CLG}}{N_{A}},\;m_{HA}=\frac{mm_{HAunit}\cdot n_{HA}}{N_{A}},\;m_{H2O}=\frac{mm_{H2Ounit}\cdot n_{H2O}}{N_{A}}. \end{array}$$

From Equation (A2), the density of the system ($\rho_{sys}$) is obtained.

(A11) $$\rho_{sys}={\left(\frac{{\mathrm{Mf}}_{CLG}}{\rho_{CLG}}+\frac{{\mathrm{Mf}}_{HA}}{\rho_{HA}}+\frac{{\mathrm{Mf}}_{H2O}}{\rho_{H2O}}\right)}^{-1}$$

From Equations (A5) and (A3), the volume fractions of the constituents are obtained.

(A12) $${\mathrm{Mf}}_{CLG}=\frac{m_{CLG}}{m_{sys}}=\frac{\rho_{CLG}V_{CLG}}{\rho_{sys}V_{sys}}=\frac{\rho_{CLG}{\mathrm{Vf}}_{CLG}}{\rho_{sys}},\;{\mathrm{Vf}}_{HA}=\frac{\rho_{sys}{\mathrm{Mf}}_{HA}}{\rho_{HA}},\;{\mathrm{Vf}}_{H2O}=\frac{\rho_{sys}{\mathrm{Mf}}_{H2O}}{\rho_{H2O}}.$$

From Equation (A3), the total mass of the system and consequently, of HA and H2O, is obtained.

(A13) $$m_{sys}=\frac{m_{CLG}}{{\mathrm{Mf}}_{CLG}}=\frac{mm_{CLG}}{{\mathrm{Mf}}_{CLG}\cdot N_{A}},\;m_{HA}={\mathrm{Mf}}_{HA}\cdot m_{sys},\;m_{H2O}={\mathrm{Mf}}_{H2O}\cdot m_{sys}.$$

Thus, from Equation (A10), the desired number of $H_{2}O$ and HA molecules are:

(A14) $$n_{HA}=\frac{m_{HA}\cdot N_{A}}{mm_{HAunit}}=\frac{mm_{HA}}{mm_{HAunit}}\;n_{H2O}=\frac{m_{H2O}\cdot N_{A}}{mm_{H2Ounit}}=\frac{mm_{H2O}}{mm_{H2Ounit}}$$

The fraction of the volume occupied by H${}_{2}$O is used for the computations of the number of ions of each type (chloride and sodium). This can be determined in two different ways:

(A15) $$V_{H2O}={\mathrm{Vf}}_{H2O}\cdot V\;\mathrm{or}\;V_{H2O}=\frac{mm_{H2Ounit}\cdot n_{H2O}}{rho_{H2O}\cdot N_{A}}\cdot 10^{24}.$$

A total of 132 chloride ions and 0 sodium ions (accounted for in Table 1) were added to all the devised models. They were calculated such that the system exhibits a salt concentration of 0.16 mol/L.

## Appendix E. HA Structure and FF Files

The IFF contains in its database Material Studios *.car* and *.mdf* files for HA. However, to use Packmol and NAMD to devise and simulate the Bone Fiber model, files in the PDB and PSF format are required.

The msi2namd tool, a modification of msi2lmp Version 3.9.6 developed at the Heinz laboratory from IFF [49], was used to convert the hap_unit_cell.car and hap_unit_cell.mdf files from the IFF model database to the PDB and PSF formats.

Note that if only one HA UC, i.e., one HA molecule, is needed, the PSF file created with msi2namd would be useful for further simulations. However, since the goal was to add several HA and $H_{2}O$ molecules to a box with CLG molecules, i.e., to devise the Bone Fiber, a CHARMM topology file was required so that the VMD psfgen plugin can be used to generate a PSF file for models with several HA UCs, i.e., several HA molecules. The CHARMM topology file for HA was manually created and tested against the PSF file generated using msi2namd. The CHARMM parameter file for HA was taken from the IFF database.

The used HA structure and FF files are available in the Supplementary Materials.
