# Supplementary material for: Devising Bone Molecular Models at the Nanoscale: From Usual Mineralized Collagen Fibrils to the First Bone Fibers Including Hydroxyapatite in the Extra-Fibrillar Volume
Source: Materials (Basel). 2022 Mar 19;15(6):2274. doi: 10.3390/ma15062274 (PMC8955169; doi:10.3390/ma15062274)
Supplement: Supplementary file 1 [file materials-15-02274-s001.zip › Supplementary_Materials/3-Bone_Fiber/1_Align/la1.0/la.html]

The Hume Linear Algebra Tcl Package


# The Hume Linear Algebra Tcl Package, La

 

## Feature Summary

 

The
package consists of Tcl procedures for the manipulation of vectors and
matrices.  The functionality spans
scaling, normalization, concatenation by rows or columns, subsetting by rows or
columns, formatted printing, transpose, dot product, matrix multiplication,
solution of linear equation sets, matrix inversion, eigenvalue/eigenvector
solutions,  singular value
decomposition, and solution of linear least squares.  The singular value decomposition can be used to perform the
principle components analysis of multivariate statistical process control and
avoid the possibly ill-conditioned multiplication of the XXt  matrix of 
observations.

 

The user can
mix vectors and arrays in linear algebra operations.  The logic does reasonable conversion of types.  Sophisticated operations such as evaluating
a custom procedure against each element of a matrix are easily possible.  Data is represented as ordinary Tcl list
variables so that the usual commands of Tcl are useable, and efficient access
to the data elements using compiled extensions is possible.

 

This
document and the package are ©Copyright 2001, Hume Integration Software.  You may use the package without licensing
fees according to the License Terms.

# User Guide

 

## Obtaining the Package

 

The
package may be obtained from the webpage http://www.hume.com/la. 
The package uses the "string is" Tcl command which was new
with Tcl 8.1.  So unless you are
comfortable making minor changes to the Tcl source code, you should only use
the package with Tcl version 8.1 and newer. 
The version 1.0 package only consists of text files, there are no binary
files.  The only difference between the
Windows archive and the POSIX archive is the difference in line feed / carriage
return characters at the end of each line of text.  The package is distributed as zip archives.  You are expected to use your own software to
unpack the archive files. 

## Installation

 

The
package is installed by extracting from the zip archive file, the package
directory and files.  The package
directory should be added into the base directory of the Tcl runtime libraries.  So if your Tcl runtime library directory is
/usr/local/lib/tcl8.3, copy or extract the la1.0 directory and files to
/usr/local/lib so that you have the directory /usr/local/lib/la1.0 and the
package files are found as /usr/local/lib/la1.0/\*.\* .  The Tcl code should be compatible with Tcl version 8.1 and later
for any architecture, but we have only tested it with Tcl 8.3.   You can run the package regression tests by
navigating to the package directory and sourcing the file “la.test”.

 

## Runtime Usage

 

The software
is found and made useable by your Tcl/Tk interpreter after you execute “package
require La”.  You typically import the
package procedure names into your global namespace to save yourself from having
to qualify the procedure names using the package name.  So typical initialization code looks like:

 

% package require La

1.0

% namespace import La::\*

 

## Operand Formats

The
package uses ordinary Tcl variables to represent scalars, vectors, and
arrays.  This means that the ordinary
Tcl commands are useable.  For example,
the Tcl set command can be used to copy a matrix:

 

set A [mident 5]  ;# A is assigned a 5x5 identity matrix

set B $A          ;# B is assigned the value of A

 

Tcl list
formats are used for better performance than arrays and for better
compatibility with C/C++ code.  
Dimension information is added in a simple way at the front of the list,
for vectors, matrices, and higher dimensional variables.

 

### Scalar Format

If the
list length of a variable is 1 the operand is a scalar, eg.,
"6.02e23".  The Tcl command
llength can be used to determine the list length.

### Vector Format

 

A vector of length N has the
representation as a Tcl list of:

 

2 N 0 v[0] v[1] v[2] ... v[N-1]

 

The first
element in the list, the value 2, signifies that there are two additional
elements of size information; the number of rows, and the number of
columns.  When the number of columns is
0, the operand is defined to be a one dimensional vector.  So the Tcl list sequence of {2 4 0 1 2 3 4}
represents the vector {1 2 3 4}.  A vector of length N has the same number of
elements as an Nx1 or 1xN matrix so they can be efficiently converted in place,
and indexing operations are simplified.

 

The index into the
underlying Tcl list for vector v[i] is

 

set index [expr {3 + $i}]

 

A vector
of length N is promoted to an N rows by 1 column matrix if used in an operation
where a matrix argument is expected.

 

The
transpose of a vector of length N is a 1 row by N columns matrix.

### Matrix Format

 

The Tcl
list data used to represent two dimensional matrix a[R,C], has the format:

 

2 R C a[0,0] a[0,1] a[0,2] ...
a[0,C-1] \

a[1,0] a[1,1] ... a[1,C-1] \

... a[R-1,C-1] 

 

where R is
the number of rows, and C is the number of columns, and the backslash character
has been used to indicate that the multiple lines of text constitute a single
list sequence.  The format itself is
independent of whether the user thinks that indexing starts from 0 or 1.  In Tcl, indexing of lists always starts with
0 so we have chosen to consistently start indexing with 0.

 

For a
valid matrix, both the number of rows and the number of columns are positive
integers.  The data elements following
the {2 R C} dimension information can also be thought of as first row, second
row, …, last row.  The index into the
underlying Tcl list for a[i,j] is:

 

set index [expr {3 + $i \* $C +
$j}]

 

### Higher Dimensions

 

If the
first element in a Tcl list is > 2, the package assumes the list represents
a higher dimensional operand.  Logic for
higher dimension operands is not currently part of the package. 

 

The
candidate structure for 3-D data is:

 

3 P R C a[0,0,0] a[0,0,1]
a[0,0,2] ... a[0,0,C-1] ... a[P-1,R-1,C-1]

 

where
P=planes, R=rows, C=columns.  An
intuitive view is that the data is multiple 2-D planes of rows and columns
listed in order from the 0 plane to the P-1 plane. 

 

The index
into the underlying Tcl list for a[i,j,k] is

 

set index [expr 4 + $i\*$R\*$c +
$j\*$c + $k]

 

### Operand Examples

 

set pi 3.1415            ;# a scalar

set v {2 1 0 3.1415}   
;# v[1] with value 3.1415

# vectors should use the first dimension of 2

set v {2 0 1 3.1415}   
;# error - vector should use first dimension only

set v {2 3 0 1 2 3 4}  
;# error - vector has an additional element

set m {2 2 3 1 2 3 4 5 6}  ;# m[2,3]

show $m

1 2 3

4 5 6

show [transpose $m]

1 4

2 5

3 6

 

## Argument Passing By Value and By Reference

There are
typically two procedures defined for an algorithm.  A plainly named procedure such as "transpose", and
another procedure with the suffix **\_br** such as
"transpose\_br".  The plainly
named procedures expect their data arguments to be passed by value, which is
the usual argument passing convention with Tcl programming.  The plain calls are designed for ease of
interactive use, and in general have been coded to perform more conversion of
arguments, more error checking, and to trade off efficiency for
convenience.  The **\_br** procedures
are intended for efficent use, with the **\_br** indicating that data
arguments are passed "By Reference" to avoid copying the data.  In Tcl, to pass by reference means that the
caller has the data in a named variable, and the caller passes the name of the
variable instead of a copy of the data. 
You can see that passing by reference is more efficient for larger
vectors and arrays.  The **\_br**
procedures in general assume that the data arguments have the correct
structure, so the caller may need to use the **promote**, **demote**, and
**transpose** calls to prepare arguments for a **\_br** call.

 

## A Note on Performance

 

Number
crunching in Tcl?  The conventional
wisdom is that computationally intensive tasks such as numerical analysis are
high on the list of things that you should not do in a scripting language.   It is true that you can obtain better
performance using a traditional compiled language such as Fortran, or C
code.   But nowadays, the performance
gain that you will obtain by switching languages is similar to the performance
gain you will see between a new computer and a computer from two years
ago.   In many situations, the actual
time spent processing numbers is a small portion of the overall project.  The time it takes the researcher to explore
his data, decide on the desired approach, and develop the software for his
desired analysis is the only performance time that really matters.   Here, a high-level development environment
such as Tcl or MATLAB wins over C++ or Java hands down.   Actual performance numbers for the Tcl
package are nothing to be ashamed about.  
Using a 900Mhz Pentium III notebook computer, the following times were
obtained for inverting an NxN matrix using Gauss elimination with partial
pivoting.   This is something of a worst
case since most numerical algorithms avoid the direct inversion of a full
matrix.  Often a major performance
improvement is made by choosing a better algorithm.

 

 

|  |  |
| --- | --- |
| Inversion of an NxN matrix.     Development time for the following timed benchmark: 15 seconds.  It worked properly on the first attempt.  The flush and update commands are not needed except to show progress before completion.  The benchmark:     % source la.tcl  % namespace import La::\*  % foreach n {10 20 50 100} {     puts [time {msolve [mdingdong $n] [mident $n]}]     flush stdout     update   } | |
| **Matrix Dimension (N)** | **Inversion Time (seconds)** |
| 10 | 0.020 |
| 20 | 0.140 |
| 50 | 1.973 |
| 100 | 15.43 |

 

Here are
some tips for efficient coding.  In Tcl
the expr command is used for the evaluation of
numeric expressions.  You should almost
always surround the arguments to **expr** with braces to prevent the Tcl
interpreter from substituting the arguments. 
The **expr** command itself is able to substitute variable references
directly and avoid re-interpreting their string representation.  For example:

 

set index [expr {3 + $i \* $C +
$j}]   ;# good example

set index [expr 3 + $i\*$C +
$j]       ;# less efficient example

 

The
Windows NT/Windows 2000 Tk console does not perform very well when large text
strings are displayed.  You may find it
preferable to print results by separate rows for large output sets, or to write
large output results to a file.  Another
alternative to the Windows Tk console, is to use tclsh83.exe from a command
window.

 

Tcl
maintains an internal binary representation for variable values, and computes a
string representation for a variable value only when needed.   If your logic is inconsistent and sometimes
treats a variable as a string, and sometimes treats it as a list of numbers,
you can cause inefficient “shimmering” of the internal representation.  For best efficiency use list oriented
commands to manipulate your vector and matrix variables, and don’t mix string
oriented commands.  For example:

 

      lappend m
$newvalue      ;# good – lappend is a list
oriented procedure

      append m
" $newvalue"    ;# bad, this
causes conversion to a string

 

The last
tip is that it is very easy to use the built-in **time**
command to analyze actual performance of your code. 

 

## A PCA Example

 

The
package contains a worked Principle Components Analysis problem based on
Section 6.5 of the SEMATECH/NIST Statistics Handbook, http://www.itl.nist.gov/div898/handbook.

 

See the
file NIST.tcl.

# Reference

 

## Alphabetical Procedure Table

 

|  |  |
| --- | --- |
| **Procedure Name(s)** | **Description** |
| demote, demote\_br | Demote an Nx1 or 1xN matrix to a vector[N].  Demote a vector[1] to a scalar.  Call twice to demote a 1x1 matrix to scalar. |
| dim, dim\_br | Return the dimension of the argument,  and to some degree verify a proper format. |
| dotprod, dotprod\_br | Dot Product = sum over i, Ai \* Bi  Can work columns or rows in matrices because indexing increments are optional arguments. |
| join\_cols, join\_cols\_br | Combine vector or matrices as added columns. |
| join\_rows, join\_rows\_br | Combine vector or matrices as added rows. |
| lassign, lassign\_br | Replace a single value in a Tcl list. |
| madd | Computes a new matrix or vector from the addition of corresponding elements in two operands. |
| madjust | Apply a scale factor and offset to the operand’s elements. |
| mat\_binary\_op, mat\_binary\_op\_br | Perform binary operations such as addition on corresponding elements of vectors or matrices. |
| mat\_unary\_op, mat\_unary\_op\_br | Perform unary operations on elements like scaling. |
| mathprec | Returns the smallest number epsilon, such that 1+epsilon > 1.  Also reports on the machine radix, digits, and rounding behavior |
| mcols, mcols\_br | Return the number of columns in a matrix or vector.  You can use mrows and mcols to keep your software isolated from the details of the data representation. |
| mdiag | Creates a diagonal matrix from a vector, an Nx1 matrix, or a 1XN matrix. |
| mdingdong | Create the Ding Dong test matrix, a Cauchy matrix that is represented inexactly in the machine, but very stable for inversion by elimination methods. |
| mdiv | Computes a new matrix or vector from the division of corresponding elements in two operands. |
| mevsvd, mevsvd\_br | Solve for the eigenvectors and eigenvalues of a real symmetric matrix by singular value decomposition. |
| mhilbert | Create the Hilbert test matrix which is notorious for being  ill conditioned for eigenvector/eigenvalue solutions. |
| mident | Create an identity matrix of order N. |
| mlssvd | Linear least squares solution for over-determined linear equations using singular value decomposition. |
| mmult, mmult\_br | Ordinary matrix multiplication. |
| mnorms, mnorms\_br | Compute the means and standard deviations of each column. |
| mnormalize, mnormalize\_br | Normalize each column by subtracting the corresponding mean and then dividing by the corresponding standard deviation. |
| moffset | Add a constant to the elements of an operand. |
| mprod | Computes a new matrix or vector from the product of corresponding elements in two operands. |
| mrange, mrange\_br | Return a subset of selected columns, selected rows as a new matrix.  Also can be used to reverse the ordering when the start index > end index. |
| mround, mround\_br | Round off elements in a matrix if they are close to integers. |
| mrows, mrows\_br | Return the number of rows in a matrix or vector.  You can use mrows and mcols to keep your software isolated from the details of the data representation. |
| mscale | Multiply each element in an operand by a factor. |
| msolve, msolve\_br | Solve the matrix problem Ax = p for x, where p may be multiple columns.  When p is the identity matrix, the solution x, is the inverse of A.  Uses Gauss elimination with partial pivoting. |
| msub | Computes a new matrix or vector from the subtraction of corresponding elements in two operands. |
| msum, msum\_br | Compute the sums of each column, returning a vector or scalar result.  Call twice to get the total sum of columns and rows (set total [msum [msum $a]]). |
| msvd | Perform the Singular Value Decomposition of a matrix. |
| promote, promote\_br | Promote a scalar or vector to an array.  Vector[N] is promoted to an Nx1 array. |
| show, show\_br | Return a formatted string representation for an operand.  Options allow for specify the format of numbers, and the strings used to separate column and row elements. |
| transpose, transpose\_br | Performs the matrix transpose, exchanging [i,j] with [j,i].  A vector is promoted to a 1xN array by transpose. |
| vdiag, vdiag\_br | Create a vector from the diagonal elements of a matrix. |
| vtrim, vtrim\_br | For a vector or matrix operand, just return the actual data elements by trimming away the dimension and size data in the front |

 

 

### demote x

### demote\_br name\_x\_in {name\_out {}}

 

Demote an Nx1 or 1xN matrix to a vector[n].  Demote a vector[1] to a scalar.  Call twice to demote a 1x1 matrix to
scalar.  For the \_br procedure, the
default output destination is to overwrite the input.

 

### dim x

### dim\_br name\_x

 

Return
the dimension of the argument,  and to
some degree verify a proper format.  Returns
0 for a scalar, 1 for a vector, 2 for a matrix, and an empty string, {}, for an
empty input value.  Most improper
formats will result in an error.

### dotprod a b {N {}} {a0index 3} {b0index 3} {ainc 1} {binc 1}

### dotprod\_br a\_name b\_name {N {}} {a0index 3} {b0index 3} {ainc 1} {binc 1}

Perform the dot product of two operands, **a**
and **b**, returning a scalar result. 
The default arguments will correctly process vector and conformable 1xN
or Nx1 matrices.  The argument **N**
is the number of element pairs to multiply when computing the sum of pair
products.  The **a0index** and **b0index**
optional arguments are the offset of the first element in the **a** and **b**
operands, respectively.  The **ainc**
and **binc** optional arguments are the index increment values to be added
to the indexes into **a** and **b** to obtain subsequent elements.

 

### join\_cols a b

### join\_cols\_br {a\_in b\_in {c\_out {}}

Combine vector or matrices as added columns,
returning a matrix result.  The
arguments must have the same number of rows. 
The default output for the **join\_cols\_br** call is to overwrite the**a** input.

 

### join\_rows a b

### join\_rows\_br {a\_in b\_in {c\_out {}}

Combine vector or matrices as added rows, returning
a matrix result.  The arguments must
have the same number of columns.  The
default output for the **join\_rows\_br** call is to overwrite the **a**
input.

### lassign list index value

### lassign\_br listname index value

Replace a single element in a Tcl list.  There is conditional logic in the package so that the **lassign\_br**
could be replaced by a C code version that would be able to update the list
directly without copying the data the way **lreplace**
does.

### madd a b

This procedure uses **mat\_binary\_op** to return a matrix or
vector result from the addition of corresponding elements in two operands.

### madjust a scale offset

This procedure uses **mat\_unary\_op** to return a matrix or
vector where the elements have been multiplied by a common factor, **scale**,
and then the **offset** value has been added.

 

### mat\_binary\_op a b {op +}

### mat\_binary\_op\_br a\_in b\_in op {c\_out {}}

This procedure is used to execute binary operations
such as addition on corresponding elements of vector or matrix operands.  The default output for the \_br call is to
overwrite the **a** input.

### mathprec {puts puts}

Returns the smallest number epsilon, such that
1+epsilon > 1.  Also reports on the
machine radix, digits, and rounding behavior, by using $puts as a command with
a single string argument, of the format “radix=2.0 digits=53
epsilon=2.22044604925e-016 method=truncation”. 
The default value of the puts argument causes the string to be printed
at the console.

### mcols m

### mcols\_br m\_name

Return the number of columns in a matrix or
vector.  You can use mrows
and **mcols** to keep your software isolated from the details of the data
representation.

 

### mdiag v

Creates a diagonal matrix from a vector, an Nx1 matrix, or a 1xN
matrix.

### mdingdong N

Create the Ding Dong test matrix of size NxN.  The matrix is a Cauchy matrix that is represented
inexactly in the machine, but very stable for inversion by elimination
methods.  Created by Dr. F. N. Ris
(Nash, 1979).

### mdiv a b

Uses **mat\_binary\_op** to compute a new matrix or vector from
the division of corresponding elements in two operands. 

### mevsvd A {epsilon 2.3e-16}

### mevsvd\_br A\_in\_out evals\_out {epsilon 2.3e-16}

Solve for the eigenvectors and eigenvalues of a real symmetric
matrix by singular value decomposition. 
The eigenvectors of the solution are returned as the columns of A.  The epsilon argument is expected to be the
value returned by the **mathprec** procedure for your platform.

### mhilbert N

Create the Hilbert test matrix which is notorious for being  ill conditioned for eigenvector/eigenvalue
solutions. (Nash, 1979)

### mident N

Create an identity matrix of order N.

### mlssvd A y {q 0.0} {puts puts} {epsilon 2.3e-16}

Solve the linear least squares solution for over-determined linear
equations using singular value decomposition. 
Solves the problem **A**[m,n]**x**[n] ~ **y**[m] for **x**[n]
where each row of **A** is a set of dependent variable values, **x**[n]
is the vector of independent variables, and **y**[m] is the set of dependent
values such as measured outcome values. 
The first column of **A** is usually all ones to compute a constant
term in the regression equation.  The value
**q** is specified such that singular values less than **q** are treated
as zero.  Typically a judgment is made
as to what variation is significant, and what is just noise.  The significance level varies by
application.  For example, a small value
of **q** might be appropriate to detect a new planet from orbital data,
whereas larger values would be used to regress socio-economic statistics.  The default value of the **puts**
argument causes the singular values to be printed at the console after the
matrix is factored.  The **epsilon**
argument is expected to be the result of the **mathprec** procedure for your
platform.

### mmult A B

### mmult\_br name\_A name\_B C\_out

Ordinary matrix multiplication, **A**[p,q] x **B**[q,r] = **C**[p,r].  Vector arguments are  promoted to Nx1 arrays, so chances are if
you are using one as a left operand you probably intend to use the transpose of
it (1xN), which is easily done using the transpose
procedure.  The **mmult** procedure
returns the value of the matrix product. 
The **mmult\_br** procedure writes the matrix product into the
variable whose name is **C\_out**. 
The variable name **C\_out** should specify a different variable than
either of the input variables specified by **name\_A** or **name\_B**.

### mnorms a

### mnorms\_br name\_a means\_out sigmas\_out

Compute the means and standard deviations of each column of the
input matrix.  Vector results are
returned.  If the number of rows is less
than 2, the standard deviations cannot be calculated and an error is returned.

### mnormalize a means sigmas

### mnormalize\_br name\_a means\_in sigmas\_in {c\_out {}}

Normalize each column of a matrix by subtracting the corresponding
mean and then dividing by the corresponding standard deviation.  The default output matrix for **mnormalize\_br**
is to overwrite the input matrix specified by **name\_a**.

### moffset a delta

Uses **mat\_unary\_op** to add a scalar constant to the elements
of an operand.  The modified operand is
the returned result.

### mprod a b

Uses **mat\_binary\_op** to compute a new matrix or vector from the
multiplicative product of corresponding elements in two operands.

### mrange m col\_start col\_last {row\_start 0} {row\_last end}

### mrange\_br name\_m\_in c\_out col\_start col\_last {row\_start 0} {row\_last end}

Returns a subset of the selected columns and selected rows of a
matrix as a new matrix.  Column and row
indexing begin with 0.  The token **end**may be used to specify the last row or column.  The default values for row selection will return all rows.  The rows and columns are read and copied
from the input matrix in the order of start to last.  The selection result includes rows and columns with index values
equal to the start indexes and proceeds to the last indexes, including all rows
and columns with indexes between the start and last values and equal to the start
and last values.  If the start index is
less than the last index for row or column selection, the result matrix is
created with the row or columns in reverse order.  This is a feature, not a bug. 

% set m {2 2 3 1 2 3 4 5 6}

% show $m

1 2 3

4 5 6

% show [mrange $m 0 1]

1 2

4 5

% show [mrange $m end 0]

3 2 1

6 5 4

% show [mrange $m 1 1 0 0]

2

### mround a {epsilon 1.0e-8}

Uses **mat\_unary\_op** to round-off to the nearest
integer the elements of a matrix or vector which are within epsilon of an
integer value.

% show $m %12.4g

           1  
1.388e-016   8.327e-017  -2.776e-017

  1.388e-016           
1  -5.551e-017   -1.11e-016

  8.327e-017 
-5.551e-017            1  -5.551e-017

 -2.776e-017  
-1.11e-016  -5.551e-017            1

% show [mround $m]

1.0 0.0 0.0 0.0

0.0 1.0 0.0 0.0

0.0 0.0 1.0 0.0

0.0 0.0 0.0 1.0

### mrows m

### mrows\_br m\_name

Return the number
of rows in a matrix or vector.  You can
use **mrows** and mcols to keep
your software isolated from the details of the data representation.

### mscale a scalefactor

Uses **mat\_unary\_op** to multiply each element in
a matrix or vector operand, **a**, by the scalar **scalefactor**.

### msolve A p

### msolve\_br Ap\_in {tolerance 2.3e-16}

Solves a system of linear
equations, **Ax** = **p**, for **x**, by brute force application of
Gauss elimination with partial pivoting. 
When **p** is the
identity matrix, the solution **x**, is the inverse of matrix **A**.The **msolve\_br** procedure accepts as input the
columnwise concatenation of **A** and **p**, and overwrites the **p**
columns with the solution columns **x**.  
The **msolve** procedure accepts **A** and **p** as separate
arguments, and returns the solution data directly.  The **epsilon**
argument is expected to be the result of the **mathprec** procedure for your
platform.

### msub a b

Uses **mat\_binary\_op** to compute a new matrix or vector from
the subtraction of corresponding elements in two operands.

### msum a

### msum\_br name\_a sums\_out

Compute the sums of each column of a matrix or
vector, returning a vector or scalar result. 
Call twice to get the total sum of columns and rows (set total [msum
[msum $a]]).

### msvd a

### msvd\_br name\_a\_in\_U\_out S\_out V\_out {epsilon 2.3e-16}

Perform the Singular Value Decomposition of a
matrix.

This factors matrix A into (U)(S)(Vtrans) where

    A[m,n] is the original
matrix

    U[m,n] has orthogonal columns (Ut)(U) =   (1(k)

       and multiplies to an identity matrix     ...

       supplemented with zeroes if needed        0(n-k))

    V[n,n] is orthogonal  
(V)(Vtran) = [mident $n]

       V contains the eigenvectors aka the principal components

    S is diagonal with the positive singular values of A

       Square S and divide by (m-1) to get the principal component

       eigenvalues.

   

    A[m,n]V[n,n] = B[m,n]  transforms
A to orthogonal columns, B

    B[m,n] = U[m,n]S[n,n] 

   

The **msvd** procedure outputs
formatted results to the console.  The **msvd\_br**
procedure overwrites the input matrix **a** with the **U** result
matrix.  The **epsilon** argument is expected to be the result of the **mathprec**
procedure for your platform.

### mat\_unary\_op a op

### mat\_unary\_op\_br name\_a op {name\_c\_out {}}

Perform unary operations on operand elements like scaling.  The default output of the **mat\_unary\_op\_br**
procedure is to overwrite the input specified by **name\_a**.  The logic sweeps through the matrix or
vector, and evaluates the concatenation of $**op** with the operand
element.  For example, if the value of **op**
is “expr 0.5 \*” the result would be to multiply each element by 0.5.  You can define your own procedures, and pass
the procedure name as the **op** argument.

### promote x

### promote\_br name\_x {name\_out {}}

Promote a scalar or vector to an array.  Vector[N] is promoted to an Nx1 array.  Calling promote with a matrix argument is all right; the result
is the matrix unchanged.  The default
output of **promote\_br** is to overwrite the input.

### show x {format {}} {col\_join { }} {row\_join \n}}

### show\_br name\_in {name\_out {}} {format {}} {col\_join { }} {row\_join \n}

Return a formatted string representation for an operand.   Options allow for specify the format of
numbers, and the strings used to separate column and row elements.  The optional **format** argument is a
specification string which is applied to convert each element using the Tcl
format command. 
The format command is very is similar to the C code sprintf
command.  

 

set m {2 2 3 1 2 3 4 5 6}

% show $m %6.2f

  1.00   2.00   3.00

  4.00   5.00   6.00

% show $m {} , \;

1,2,3;4,5,6

### transpose x

### transpose\_br name\_x {name\_out {}}

Performs the matrix transpose, exchanging [i,j] with
[j,i].  A vector is promoted to a 1xN
array by transpose.  The default output
of the **transpose\_br** procedure is to overwrite the input data named by **name\_x**.

### vdiag m

### vdiag\_br m\_name\_in v\_out

Create a vector from the diagonal elements of a
matrix.

### vtrim x

### vtrim\_br name\_x {name\_out {}}

For a vector or matrix operand, just return the
actual data elements by trimming away the dimension and size data in the front
of the underlying Tcl list representation. 
The default output of the **vtrim\_br** procedure is to overwrite the
input data named by **name\_x**.

# About the Author

This package has been developed by Edward C. Hume, III
PhD.  Dr. Hume has been interested in
numerical methods since the early 1980’s when his doctoral research at MIT
involved a comparison of Finite Element and Boundary Element methods for moving
boundary problems.  In recent years he
has been applying univariate and multivariate Statistical Process Control
techniques in his consulting work.  Dr.
Hume is the founder of Hume Integration Software, a software product and
consulting company, with a focus on Computer Integrated Manufacturing in the
Semiconductor and Electronics industries.

 

Hume Integration's flagship product is the **Distributed Message
Hub (DMH) Application Development Package**, a cohesive and synergistic set
of tools that extends the Tcl/Tk programming environment. The package includes
an in-memory SQL database that has subscription capability, comprehensive
support for equipment interfaces using SECS, serial, or network protocols, and
high-level facilities for interprocess communication. Applications can easily
share data and logic by exchanging Tcl and SQL messages which are efficiently
processed by the extended interpreter. This toolset is offered for Windows
2000/NT and major POSIX platforms including HP-UX, Linux, AIX, and SOLARIS. The
toolset is in 7x24 use in dozens of factories located in the United States,
Malaysia, Korea, Japan, China, Hong Kong, Singapore, Taiwan, Mexico, France,
and Scotland.  Feel free to visit our
website at http://www.hume.com.

 

# References

 

The more
sophisticated algorithms in this package were adapted from Nash, 1979.

 

Compact
Numerical Methods for Computers: Linear Algebra and Function Minimisation by J. C. Nash, John Wiley &
Sons, New York, 1979.

# License Terms

 

The La
package software is being distributed under terms and conditions similar to
Tcl/Tk.  The author is providing the
package software to the Tcl community as a returned favor for the value of
packages received over the years.

 

The La package software is copyrighted by Hume Integration
Software. The following terms apply to all files associated with the software
unless explicitly disclaimed in individual files.

 

The authors hereby grant permission to use, copy, modify,
distribute, and license this software and its documentation for any purpose,
provided that existing copyright notices are retained in all copies and that
this notice is included verbatim in any distributions. No written agreement,
license, or royalty fee is required for any of the authorized uses.
Modifications to this software may be copyrighted by their authors and need not
follow the licensing terms described here, provided that the new terms are
clearly indicated on the first page of each file where they apply.

 

IN NO EVENT SHALL THE AUTHORS OR DISTRIBUTORS BE LIABLE TO
ANY PARTY FOR DIRECT, INDIRECT, SPECIAL, INCIDENTAL, OR CONSEQUENTIAL DAMAGES
ARISING OUT OF THE USE OF THIS SOFTWARE, ITS DOCUMENTATION, OR ANY DERIVATIVES
THEREOF, EVEN IF THE AUTHORS HAVE BEEN ADVISED OF THE POSSIBILITY OF SUCH
DAMAGE.

 

THE AUTHORS AND DISTRIBUTORS SPECIFICALLY DISCLAIM ANY
WARRANTIES,INCLUDING, BUT NOT LIMITED TO, THE IMPLIED WARRANTIES OF
MERCHANTABILITY, FITNESS FOR A PARTICULAR PURPOSE, AND NON-INFRINGEMENT.  THIS SOFTWARE IS PROVIDED ON AN "AS
IS" BASIS, AND THE AUTHORS AND DISTRIBUTORS HAVE NO OBLIGATION TO PROVIDE
MAINTENANCE, SUPPORT, UPDATES, ENHANCEMENTS, OR MODIFICATIONS.

 

GOVERNMENT USE: If you are acquiring this software on behalf
of the U.S. government, the Government shall have only "Restricted
Rights" in the software and related documentation as defined in the
Federal Acquisition Regulations (FARs) in Clause 52.227.19 (c) (2).  If you are acquiring the software on behalf
of the Department of Defense, the software shall be classified as
"Commercial Computer Software" and the Government shall have only
"Restricted Rights" as defined in Clause 252.227-7013 (c) (1) of
DFARs.  Notwithstanding the foregoing,
the authors grant the U.S. Government and others acting in its behalf
permission to use and distribute the software in accordance with the terms
specified in this license.

 

# Document Version

Date of
last revsion $Date: 2001/11/14 13:27:35 $.
