# Supplementary material for: Devising Bone Molecular Models at the Nanoscale: From Usual Mineralized Collagen Fibrils to the First Bone Fibers Including Hydroxyapatite in the Extra-Fibrillar Volume
Source: Materials (Basel). 2022 Mar 19;15(6):2274. doi: 10.3390/ma15062274 (PMC8955169; doi:10.3390/ma15062274)
Supplement: Supplementary file 1 [file materials-15-02274-s001.zip › Supplementary_Materials/3-Bone_Fiber/1_Align/la1.0/la_cmds.html]

Linear Algebra (La) Package Contents

## Contents

La Feature Summary
  

## User Guide

Obtaining
  

Installation
  

Runtime Usage
  

Operand Formats
  

Argument Passing
  

Performance
  

PCA Example
  

## Reference

demote
  
dim
  
dotprod
  
join\_cols
  
join\_rows
  
lassign
  
madd
  
madjust
  
mat\_binary\_op
  
mat\_unary\_op
  
mathprec
  
mcols
  
mdiag
  
mdingdong
  
mdiv
  
mevsvd
  
mhilbert
  
mident
  
mlssvd
  
mmult
  
mnorms
  
mnormalize
  
moffset
  
mprod
  
mrange
  
mround
  
mrows
  
mscale
  
msolve
  
msub
  
msum
  
msvd
  
promote
  
show
  
transpose
  
vdiag
  
vtrim
  

## Author

  

## References

  

## License Terms

  

## Document Version
