# Supplementary material for: Devising Bone Molecular Models at the Nanoscale: From Usual Mineralized Collagen Fibrils to the First Bone Fibers Including Hydroxyapatite in the Extra-Fibrillar Volume
Source: Materials (Basel). 2022 Mar 19;15(6):2274. doi: 10.3390/ma15062274 (PMC8955169; doi:10.3390/ma15062274)
Supplement: Supplementary file 1 [file materials-15-02274-s001.zip › Supplementary_Materials/3-Bone_Fiber/1_Align/la1.0/la_download.html]

Tcl Linear Algebra (La) Package Download

## Linear Algebra Package Download

La package, version 1.01, for Windows (text files have CR/LF line delimiters)
  

la101win.zip
  

La package for POSIX (text files have LF line delimiters)  

la101psx.zip

### Version History

Version 1.01 differs from version 1.0 only in the renaming of
Hume Integration Services to Hume Integration Software.

---

The Hume Integration Software Home Page.
