# Supplementary material for: Devising Bone Molecular Models at the Nanoscale: From Usual Mineralized Collagen Fibrils to the First Bone Fibers Including Hydroxyapatite in the Extra-Fibrillar Volume
Source: Materials (Basel). 2022 Mar 19;15(6):2274. doi: 10.3390/ma15062274 (PMC8955169; doi:10.3390/ma15062274)
Supplement: Supplementary file 1 [file materials-15-02274-s001.zip › Supplementary_Materials/3-Bone_Fiber/1_Align/la1.0/NIST.html]

NIST PCA Example

## NIST PCA Example

```
# A Principal Components Analysis example
#
# walk through the NIST/Sematech PCA example data section 6.5
# http://www.itl.nist.gov/div898/handbook/
#
# This file is part of the Hume La Package
# (C)Copyright 2001, Hume Integration Software
#
# These calculations use the Hume Linear Algebra package, La
#
package require La
catch { namespace import La::*}


# Look at the bottom of this file to see the console output of this procedure.

proc nist_pca {} {
    puts {Matrix of observation data X[10,3]}
    set X {2 10 3 7 4 3 4 1 8 6 3 5 8 6 1 8 5 7 7 2 9 5 3 3 9 5 8 7 4 5 8 2 2}
    puts [show $X]
    puts {Compute column norms}
    mnorms_br X Xmeans Xsigmas	
    puts "Means=[show $Xmeans]"
    puts "Sigmas=[show $Xsigmas %.6f]"
    puts {Normalize observation data creating Z[10,3]}
    puts [show [set Z [mnormalize $X $Xmeans $Xsigmas]] %10.6f]\n
    puts {Naive computation method that is sensitive to round-off error}
    puts {ZZ is (Ztranspose)(Z) }
    puts "ZZ=\n[show [set ZZ [mmult [transpose $Z] $Z]] %10.6f]"
    puts {the correlation matrix, R = (Zt)(Z)/(n-1)}
    puts "R=\n[show [set R [mscale $ZZ [expr 1/9.0]]] %8.4f]"
    puts {Continuing the naive example use R for the eigensolution}
    set V $R
    mevsvd_br V evals
    puts "eigenvalues=[show $evals %8.4f]"
    puts "eigenvectors:\n[show $V %8.4f]\n"
    puts "Good computation method- perform SVD of Z"
    set U $Z
    msvd_br U S V
    puts "Singular values, S=[show $S]"
    puts "square S and divide by (number of observations)-1 to get the eigenvalues"
    set SS [mprod $S $S]
    set evals [mscale $SS [expr 1.0/9.0]]
    puts "eigenvalues=[show $evals %8.4f]"
    puts "eigenvectors:\n[show $V %8.4f]"
    puts "(the 3rd vector/column has inverted signs, thats ok the math still works)"
    puts "\nverify V x transpose = I"
    puts "As computed\n[show [mmult $V [transpose $V]] %20g]\n"
    puts "Rounded off\n[show [mround [mmult $V [transpose $V]]]]"
    #puts "Diagonal matrix of eigenvalues, L"
    #set L [mdiag $evals]
    #
    
    puts "\nsquare root of eigenvalues as a diagonal matrix"
    proc sqrt x { return [expr sqrt($x)] }
    set evals_sr [mat_unary_op $evals sqrt]
    set Lhalf [mdiag $evals_sr]
    puts [show $Lhalf %8.4f]

    puts "Factor Structure Matrix S"
    set S [mmult $V $Lhalf]
    puts [show $S %8.4f]\n
    set S2 [mrange $S 0 1]
    puts "Use first two eigenvalues\n[show $S2 %8.4f]"
    # Nash discusses better ways to compute this than multiplying SSt
    # the NIST method is sensitive to round-off error
    set S2S2 [mmult $S2 [transpose $S2]]
    puts "SSt for first two components, communality is the diagonal"
    puts [show $S2S2 %8.4f]\n


    # define reciprocal square root function
    proc recipsqrt x { return [expr 1.0/sqrt($x)] }
    # use it for Lrhalf calculation
    set Lrhalf [mdiag [mat_unary_op $evals recipsqrt]]

    set B [mmult $V $Lrhalf]
    puts "Factor score coefficients B Matrix:\n[show $B %12.4f]"

    puts "NIST shows B with a mistake, -.18 for -1.2 (-1.18 with a typo)"

    puts "\nWork with first normalized observation row"
    set z1 {2 3 0 0.065621795889 0.316227766017 -0.7481995314}
    puts [show $z1 %.4f]

    puts "compute the \"factor scores\" from multiplying by B"
    set t [mmult [transpose $z1] $B]
    puts [show $t %.4f]
    puts "NIST implies you might chart these"

    #set T2 [dotprod $t $t]
    #puts "Hotelling T2=[format %.4f $T2]"
    
    puts "Compute the scores using V, these are more familiar"
    set t [mmult [transpose $z1] $V]
    puts [show $t %.4f]
    puts "Calculate T2 from the scores, sum ti*ti/evi"

    set T2 [msum [transpose [mdiv [mprod $t $t] [transpose $evals]]]]
    puts "Hotelling T2=[format %.4f $T2]"
 
    puts "Fit z1 using first two principal components"
    set p [mrange $V 0 1]
    puts p=\n[show $p %10.4f]
    set t [mmult [transpose $z1] $p]
    set zhat [mmult $t [transpose $p]]
    puts "  z1=[show $z1 %10.6f]"
    puts "zhat=[show $zhat %10.6f]"
    set diffs [msub [transpose $z1] $zhat]
    puts "diff=[show $diffs %10.6f]"
    puts "Q statistic - distance from the model measurement for the first observation"
    set Q [dotprod $diffs $diffs]
    puts Q=[format %.4f $Q]
    puts "Some experts would compute a corrected Q that is larger since the 
observation was used in building the model.  The Q calculation just used
properly applies to the analysis of new observations."
    set corr [expr 10.0/(10.0 - 2 -1)]  ;# N/(N - Ncomp - 1)
    puts "Corrected Q=[format %.4f [expr $Q * $corr]] (factor=[format %.4f $corr])"
    }

return
##########################################################################
Console Printout
##########################################################################
Matrix of observation data X[10,3]
7 4 3
4 1 8
6 3 5
8 6 1
8 5 7
7 2 9
5 3 3
9 5 8
7 4 5
8 2 2
Compute column norms
Means=6.9 3.5 5.1
Sigmas=1.523884 1.581139 2.806738
Normalize observation data creating Z[10,3]
  0.065622   0.316228  -0.748200
 -1.903032  -1.581139   1.033228
 -0.590596  -0.316228  -0.035629
  0.721840   1.581139  -1.460771
  0.721840   0.948683   0.676942
  0.065622  -0.948683   1.389513
 -1.246814  -0.316228  -0.748200
  1.378058   0.948683   1.033228
  0.065622   0.316228  -0.035629
  0.721840  -0.948683  -1.104485

Naive computation method that is sensitive to round-off error
ZZ is (Ztranspose)(Z) 
ZZ=
  9.000000   6.017916  -0.911824
  6.017916   9.000000  -2.591349
 -0.911824  -2.591349   9.000000
the correlation matrix, R = (Zt)(Z)/(n-1)
R=
  1.0000   0.6687  -0.1013
  0.6687   1.0000  -0.2879
 -0.1013  -0.2879   1.0000
Continuing the naive example use R for the eigensolution
eigenvalues=  1.7688   0.9271   0.3041
eigenvectors:
  0.6420   0.3847  -0.6632
  0.6864   0.0971   0.7207
 -0.3417   0.9179   0.2017

Good computation method- perform SVD of Z
Singular values, S=3.98985804571 2.88854344819 1.65449373616
square S and divide by (number of observations)-1 to get the eigenvalues
eigenvalues=  1.7688   0.9271   0.3041
eigenvectors:
  0.6420   0.3847   0.6632
  0.6864   0.0971  -0.7207
 -0.3417   0.9179  -0.2017
(the 3rd vector/column has inverted signs, thats ok the math still works)

verify V x transpose = I
As computed
                   1        -5.55112e-017         1.38778e-016
       -5.55112e-017                    1         5.55112e-017
        1.38778e-016         5.55112e-017                    1

Rounded off
1.0 0.0 0.0
0.0 1.0 0.0
0.0 0.0 1.0

square root of eigenvalues as a diagonal matrix
  1.3300   0.0000   0.0000
  0.0000   0.9628   0.0000
  0.0000   0.0000   0.5515
Factor Structure Matrix S
  0.8538   0.3704   0.3658
  0.9128   0.0935  -0.3975
 -0.4544   0.8838  -0.1112

Use first two eigenvalues
  0.8538   0.3704
  0.9128   0.0935
 -0.4544   0.8838
SSt for first two components, communality is the diagonal
  0.8662   0.8140  -0.0606
  0.8140   0.8420  -0.3321
 -0.0606  -0.3321   0.9876

Factor score coefficients B Matrix:
      0.4827       0.3995       1.2026
      0.5161       0.1009      -1.3069
     -0.2569       0.9533      -0.3657
NIST shows B with a mistake, -.18 for -1.2 (-1.18 with a typo)

Work with first normalized observation row
0.0656 0.3162 -0.7482
compute the "factor scores" from multiplying by B
0.3871 -0.6552 -0.0608
NIST implies you might chart these
Compute the scores using V, these are more familiar
0.5148 -0.6308 -0.0335
Calculate T2 from the scores, sum ti*ti/evi
Hotelling T2=0.5828
Fit z1 using first two principal components
p=
    0.6420     0.3847
    0.6864     0.0971
   -0.3417     0.9179
  z1=  0.065622   0.316228  -0.748200
zhat=  0.087847   0.292075  -0.754958
diff= -0.022225   0.024153   0.006758
Q statistic - distance from the model measurement for the first observation
Q=0.0011
Some experts would compute a corrected Q that is larger since the 
observation was used in building the model.  The Q calculation just used
properly applies to the analysis of new observations.
Corrected Q=0.0016 (factor=1.4286)
```
