# Supplementary material for: Devising Bone Molecular Models at the Nanoscale: From Usual Mineralized Collagen Fibrils to the First Bone Fibers Including Hydroxyapatite in the Extra-Fibrillar Volume
Source: Materials (Basel). 2022 Mar 19;15(6):2274. doi: 10.3390/ma15062274 (PMC8955169; doi:10.3390/ma15062274)
Supplement: Supplementary file 1 [file materials-15-02274-s001.zip › Supplementary_Materials/3-Bone_Fiber/1_Align/la1.0/products.html]

Hume Integration Tcl/Tk/DMH Documentation


## Hume Integration Software

### Document Sections

Linear Algebra Package  

LA Package Download
