# Supplementary material for: Devising Bone Molecular Models at the Nanoscale: From Usual Mineralized Collagen Fibrils to the First Bone Fibers Including Hydroxyapatite in the Extra-Fibrillar Volume
Source: Materials (Basel). 2022 Mar 19;15(6):2274. doi: 10.3390/ma15062274 (PMC8955169; doi:10.3390/ma15062274)
Supplement: Supplementary file 1 [file materials-15-02274-s001.zip › Supplementary_Materials/3-Bone_Fiber/HA/INTERFACE_MD/interface_ff_1_5.zip!/INTERFACE_FF_1_5/2015_03_23_log_of_version_updates.docx]

**Interface Force Field - Version History and Updates**

| **Version** | **Date** | **Changes** |
| --- | --- | --- |
| 1.0 | 2013-01-01 | Initial as published in Langmuir |
| 1.1 | 2013-01-18 | CHARMM-INTERFACE added |
| 1.2 | 2013-04-01 | Corrected typo of interchanged well depths of oc24 and oc25 (silanol O in silica and O in gypsum) in the PCFF/OFF version and associated entries in the table of automatic equivalences |
| 1.3 | 2014-04-01 | • Utility programs updated for easier use  • Parameters added for kaolinite clay (H type hok for aluminol)  • Angle parameters of AlOH/MgOH in all clays updated for structural stability (126˚ and 45 kcal/mol; some deviation in IR accepted)  • Silica surface model database expanded for all chemistries and pH  • C3A parameters updated and models added  • C3S models added  • Hydroxyapatite models for different pH added, including nanorods and sodium phosphates  • CHARMM-INTERFACE expanded for C3S, C3A, HAP, including pH responsive surfaces of HAP  • CVFF-INTERFACE added with full CHARMM functionality + PEO  • oc5-hoc distance in PCFF changed from 0.945 to 0.929 A to accommodate hydroxide ions (oc5-hoy could be used without affecting oc5-hoc in ettringite)  • Upgrade of documentation  • Addition of anhydrite to Ca sulfates  • Release of all prior versions and their publication dates |
| 1.4 | 2014-05-01 | • Models of clay edges included (SiOH termination with possible ionization; neutral cleavage of octahedral aluminate sheet to coordinate water)  • Coordinate constraints in some clay models relieved (remainder from earlier coordinate minimization of H atoms) |
| 1.5 | 2015-03-23 | • Update of pH assignment of HAP models and of O charges in the models at pH 10 and pH 5 (-0.65e in H-O-P) |

**Known Bugs/Planned Developments**
• car_lmp_TO_pdb_psf code does not detect the absence of dihedrals (C3S, HAP) and confuses
 them with impropers (requires manual adjustment in .psf file)
• cvff_nocross_nomorse.off to be included

Clays:
• check new LJ param for Si & O to reproduce contact angles as in silica Q4/Q3 (consistent with edge models according to silica silanols); [for pub: show distinct oxygen positions and swelling]
• increase H charge in Al-OH (mont/kaol/mica) from 0.20 to 0.30 (dipole involves no lone pairs; const with C3A, ettr, boehmite, higher acidity in silica)

CHARMM-INTERFACE
• test necessity of improper definitions of terminal hydrogens (e.g. X OC24 HOY X) and remove if no problem

Gypsum and other Ca sulfates:
• force field type oc25 currently unused, only oc11 employed and appears to be working fine; resolve upon full implementation of 9-6 and 12-6 Ca sulfate parameters

Hydroxyapatite:
• refine atomic charges on nanorods at pH 5.0 (currently within ballpark and neutral, though not strictly following the model reactions of hydration and protonation), pH 14 models are exact

Incoming new compounds:
• γ-FeOOH, layered titanates, γ-AlOOH, quantum dots (Zn, Cd, Pb)(S, Se, Te)
