# Supplementary material for: Devising Bone Molecular Models at the Nanoscale: From Usual Mineralized Collagen Fibrils to the First Bone Fibers Including Hydroxyapatite in the Extra-Fibrillar Volume
Source: Materials (Basel). 2022 Mar 19;15(6):2274. doi: 10.3390/ma15062274 (PMC8955169; doi:10.3390/ma15062274)
Supplement: Supplementary file 1 [file materials-15-02274-s001.zip › Supplementary_Materials/3-Bone_Fiber/HA/INTERFACE_MD/interface_ff_1_5.zip!/INTERFACE_FF_1_5/INTERFACE_documentation_v1_5.doc]

**Interface Force Field and Surface Model Database V1.5**

***User Guide***

Hendrik Heinz, Tzu-Jen Lin, Ratan K. Mishra, Fateme S. Emami, Hadi Ramezani-Dakhel

Contact: hendrik.heinz@uakron.edu

**Table of Contents**

1. Overview	2

2. Quick Guide	3

2.1. Supported Force Fields	3

2.2. Supported Compounds	4

2.3. Utility Programs	5

3. Details of Models	5

3.1. Clay minerals	5

3.2. FCC metals	8

3.3. Silica	8

3.4. Hydroxyapatite	12

3.5. Cement minerals	14

3.6. Calcium sulfates	17

3.7. Poly(ethyleneoxide)	18

4. History of Versions	19

**1. Overview**

The INTERFACE force field enables atomistic simulations of nanostructures at the 1 to 100 nm scale in high accuracy and serves as a uniform simulation platform for inorganic, organic, and biomolecular compounds.^1^ The parameters cover minerals, metals, oxides, and are integrated in harmonic force fields for biomolecules, solvents, and polymers, including CHARMM, PCFF, and CVFF. Structural and surface properties, such as surface tensions, interface tensions, and specific adsorption energies are up to two orders of magnitude more accurate than in earlier force fields and typically also better than with DFT methods (deviations up to 500% from experiment reduced to <10%). As true for all force fields, the computational cost is million times lower than with ab-initio methods in addition, and multi-resolution combinations with local quantum mechanics as well as larger-scale coarse-grain models are possible.

The parameterization relies on true first principles of chemical understanding, different from boxed ab-initio calculations that result in less reliable and empirical force field parameters (see details in ref. ^1^). Key aspects are accurate atomic charges that describe ionic versus covalent bonding,^2^ thermodynamic consistency among all compounds through validation of interfacial properties, and interpretation of all parameters. The motivation for this approach has been the need to understand assembly of inorganic-organic and inorganic-biological interfaces at length scales of 1 to 1000 nm where imaging and experimental characterization techniques are limited, the lack of reliable force fields for inorganic compounds as well as for interfaces.^3-15^ The INTERFACE approach is applicable to all compounds across the periodic table and substantial improvements are also possible for biomolecules and soft matter (ongoing efforts).

**Selected references (* Key Development)**

(1)*	Heinz, H.; Lin, T.-J.; Mishra, R. K.; Emami, F. S. *Langmuir* **2013**, *29*, 1754.

(2)*	Heinz, H.; Suter, U. W. *J. Phys. Chem. B* **2004**, *108*, 18341.

(3)	Heinz, H.; Castelijns, H. J.; Suter, U. W. *J. Am. Chem. Soc.* **2003**, *125*, 9500.

(4)*	Heinz, H.; Koerner, H.; Anderson, K. L.; Vaia, R. A.; Farmer, B. L. *Chem. Mater.* **2005**, *17*, 5658.

(5)	Heinz, H.; Vaia, R. A.; Farmer, B. L. *J. Chem. Phys.* **2006**, *124*, 224713.

(6)	Heinz, H.; Vaia, R. A.; Krishnamoorti, R.; Farmer, B. L. *Chem. Mater.* **2007**, *19*, 59.

(7)*	Heinz, H.; Vaia, R. A.; Farmer, B. L.; Naik, R. R. *J. Phys. Chem. C* **2008**, *112*, 17281.

(8)	Heinz, H.; Farmer, B. L.; Pandey, R. B.; Slocik, J. M.; Patnaik, S. S.; Pachter, R.; Naik, R. R. *J. Am. Chem.	Soc.* **2009**, *131*, 9704.

(9)*	Patwardhan, S. V.; Emami, F. S.; Berry, R. J.; Jones, S. E.; Naik, R. R.; Deschaume, O.; Heinz, H.; Perry, C.	C. *J. Am. Chem. Soc.* **2012**, *134*, 6244.

(10)	Jha, K. C.; Liu, H.; Bockstaller, M. R.; Heinz, H. *J. Phys. Chem. C* **2013**, *117*, 25969.

(11)*	Lin, T. Z. Force Field Parameters and Atomistic Surface Models for Hydroxyapatite and Analysis of	Biomolecular Adsorption at Aqueous Interfaces; University of Akron, PhD thesis, 2013,	<http://rave.ohiolink.edu/etdc/view?acc_num=akron1365511604>.

(12)*	Mishra, R. K.; Flatt, R. J.; Heinz, H. *J. Phys. Chem. C* **2013**, *117*, 10417.

(13)	Ramezani-Dakhel, H.; Mirau, P. A.; Naik, R. R.; Knecht, M. R.; Heinz, H. *Phys. Chem. Chem. Phys.* **2013**,	*15*, 5488.

(14)	Ruan, L.; Ramezani-Dakhel, H.; Chiu, C.-Y.; Zhu, E.; Li, Y.; Heinz, H.; Huang, Y. *Nano Lett.* **2013**, *13*, 840.

(15)*	Emami, F. S. et al. *Chem. Mater.* **2014**, *26*, DOI: 10.1021/cm500365c

**2. Quick Guide**

**Tips to Start**

•	Models are ready to use

•	Retain atomic charges and force field (atom) types for inorganic compounds – do not use automated reassignments

•	Existing organic compounds can	be typed and charged	automatically

•	Copy xxx.frc and the	xxx_templates.dat file to your force	field directory

Force fields and models are ready to use. Included are three folders:

•	FORCE_FIELDS

•	MODEL_DATABASE

•	UTILITY_PROGRAMS

**2.1. Supported Force Fields**

The **FORCE_FIELDS** folder contains the files:

•	pcff_interface.frc

•	pcff_interface_templates.dat

•	pcff_interface.off (same as above in .off format)

•	cvff_interface.frc

•	cvff_interface_templates.dat

•	charmm27_interface.prm (same as par_all27_prot_lipid.prm with added parameters).

**Pcff_interface** and **cvff_interface** are ready for use with the Discover and Forcite programs in Materials Studio by Accelrys, Inc. and with the LAMMPS program. In the .frc file, all new parameters for inorganic compounds and polyethylene oxide are identified at the beginning of each line with version and reference numbers: Versions 3.2-3.4, Refs. 13-18. Most of the new parameters can be found at the end of the respective sections for bond, angle, torsion, and nonbond parameters, following the existing PCFF parameters (exception: metal parameters supercede the previous metal parameters at the beginning of the nonbond section and some organic parameters are updated). Definitions of atom types are given in the list of atom types near the top of the file. The atom types for new compounds and the atom types for (bio)organic compounds in the original pcff have no overlap.

**Charmm27_interface** is ready for use with LAMMPS, NAMD, and CHARMM programs. Parameters for clay minerals, silica, apatites, metals, and some cement minerals have been added to the existing CHARMM 27 parameter file .prm. Remaining parameters for cement minerals, sulfates, and PEO are to be added later. The INTERFACE parameters are clearly identified at the end of the respective section for portability to newer CHARMM versions. Building input files for inorganic compounds (.pdb/.psf), as part of interfaces with water and biomolecules, currently relies on first obtaining LAMMPS input and subsequent conversion to CHARMM using the programs in the UTILITY_PROGRAMS folder. This route is easier than the use of topology files and psfgen for minerals with periodic covalent bonds such as clay minerals and silica. The use of topology files and psfgen would require manual adjustment of atom coordinates. Input files for biomolecules and water, as part of interfaces with inorganic compounds, can be prepared as usual using topology files and psfgen, followed by merging with the .pdb/.psf file pair for the inorganic structure using the merge_pdb_psf_files program in the UTILITY_PROGRAMS folder.

The force field parameters for many compounds can also be implemented in other force fields such as **AMBER**, **OPLS-AA**, **Dreiding**, for example, fcc metals, apatite, silica, and some cement minerals (see original references).

**2.2. Supported Compounds**

The folder **MODEL_DATABASE** contains simulation-ready structure files for the following compounds and surfaces in .car/.mdf format:

•	**Clay minerals:** kaolinite, mica, different montmorillonites, pyrophyllite, including surfaces of different CEC and cation distributions according to NMR data

•	**Metals:** fcc metals Ag, Al, Au, Cu, Ni, Pb, Pd, Pt, including {111}, {100}, and {110} surfaces

•	**Silica:** bulk minerals as well as Q^2^, Q^3^, Q^4^ and mixed surfaces of different degree of ionization that represent specific pH values and nanoparticle sizes

•	**Hydroxyapatite:** bulk mineral and all possible cleavage planes for specific pH values, as well as models of nanocrystallites at different pH values

•	**Cement_minerals:** tricalcium silicate, tricalcium aluminate, ettringite, monosulfate, tobermorite 11 Å, tobermorite 14 Å, including different cleavage planes and hydrated phases

*(CVFF and CHARMM currently support tricalcium silicate and tricalcium aluminate only)*

•	**Calcium_sulfate:** calcium sulfate (anhydrite), hemihydrate and gypsum, including different cleavage planes *(Currently supported in PCFF only)*

•	**PEO:** poly(ethylene oxide) crystal and a polymer chain, including gauche effect and solubility *(Currently supported in PCFF only)*

Modifications of the existing models are possible for any size, cleavage plane, porosity, and defect distribution using the unit cells and customized models provided. All models (.car/.mdf files) contain force field types and atomic charges ready for simulations, which can be a valuable reference in preparing similar structures. Validation of the force field performance for each compound can be found in the references provided.

**2.3. Utility Programs**

The folder **UTILITY_PROGRAMS** contains three programs to prepare .pdb and .psf files for simulations using charmm27_interface.prm. The three programs convert .car/.mdf files to LAMMPS input, convert LAMMPS and .car files into .pdb and .psf format, and merge .pdb/.psf pairs for different components into one .pdb/.psf file for simulations. To improve ease of use, a simpler one-step code is anticipated in the future.

**3. Details of Models**

**3.1. Clay Minerals**

The models are supplied in .car/.mdf format and based on experimental crystal structures except for montmorillonite where no full X-Ray derived structures are available. The structures are fully charged, typed, and ready for simulations. It is recommended, however, to briefly minimize the energy of the given crystal structure with the force field before use.

**3.1.1. Kaolinite**
Model formula: [Si_2_O_4_][Al_2_O(OH)_4_], CEC=0 mmol/100g

| kaolinite_unit_cell | Unit cell (Bish, 1993) |
| --- | --- |
| kaolinite30_cell | Supercell (5x3x2) |
| kaolinite15_single_layer | Single layer (5x3x1) in rectangular cell |

**3.1.2. Muscovite Mica**
Model formula: K[Si_3_AlO_8_][Al_2_O_2_(OH)_2_], CEC=251 mmol/100g

| mica1_cell | Unit cell (Rothbauer, 1971) with stoichiometric, but arbitrarily located Si->Al charge defects [1] |
| --- | --- |
| mica15_cell | Supercell (5x3x1) with Si->Al substitution pattern according to 29Si-NMR [2] |
| mica15_single_layer | Single layer (5x3) in rectangular cell with optimized distribution of potassium ions on both sides [2] |
| mica15_double_layer | Double layer (5x3) in rectangular cell with optimized distribution of potassium ions on both sides [2] |

**3.1.2. Montmorillonite (Three Different Cation Exchange Capacities)**
Montmorillonite 0.321 (Southern Clay) − larger supercell
Model formula: (K, Na)_0.321_[Si_4_O_8_][Al_1.679_Mg_0.321_O_2_(OH)_2_], CEC=87 mmol/100g

| mont0_321_K_28_cell | Supercell (7x4x1) with optimized Al->Mg substitution pattern and K^+^ interlayer cations, crystal structure not optimized (same as mica) [4] |
| --- | --- |
| mont0_321_Na_28_cell | Same with Na interlayer cations |
| mont0_321_K_28_single_layer | Single layer (7x4) in rectangular cell with optimized K^+^ distribution on both sides |

Montmorillonite 0.333 (Southern Clay)
Model formula: (K, Na)_0.333_[Si_4_O_8_][Al_1.667_Mg_0.333_O_2_(OH)_2_], CEC=90 mmol/100g

| mont0_333_K_15_cell | Supercell (5x3x1) with optimized Al->Mg substitution pattern and K^+^ interlayer cations, crystal structure not optimized (same as mica) [4] |
| --- | --- |
| mont0_333_Na_15_cell | Same with Na interlayer cations |
| mont0_333_K_15_single_layer | Single layer (5x3) in rectangular cell with optimized K^+^ distribution on both sides |
| mont0_333_layer_2_5nm_neutral_edges_pH3 | 2.5 nm layer (5x3) with neutral SiOH and aluminate edges to coordinate water (ionization to other pH feasible) |
| mont0_333_layer_5nm_neutral_edges_pH3 | 5 nm layer (10x6) with neutral SiOH and aluminate edges to coordinate water (ionization to other pH feasible) |

Montmorillonite 0.400
Model formula: (K, Na)_0.4_[Si_4_O_8_][Al_1.6_Mg_0.4_O_2_(OH)_2_], CEC=108 mmol/100g

| mont0_400_K_15_cell | Supercell (5x3x1) with optimized Al->Mg substitution pattern and K^+^ interlayer cations, crystal structure not optimized (same as mica) [4] |
| --- | --- |
| mont0_400_Na_15_cell | Same with Na interlayer cations |
| mont0_400_K_15_single_layer | Single layer (5x3) in rectangular cell with optimized K^+^ distribution on both sides |

Montmorillonite 0.533 (Nanocor)
Model formula: (K, Na)_0.533_[Si_4_O_8_][Al_1.467_Mg_0.533_O_2_(OH)_2_], CEC=143 mmol/100g

| mont0_533_K_15_cell | Supercell (5x3x1) with optimized Al->Mg substitution pattern and K^+^ interlayer cations, crystal structure not optimized (same as mica) [4] |
| --- | --- |
| mont0_533_Na_15_cell | Same with Na interlayer cations |
| mont0_533_K_15_single_layer | Single layer (5x3) in rectangular cell with optimized K^+^ distribution on both sides |

**3.1.3. Pyrophyllite**
Model formula: [Si_4_O_8_][Al_2_O_2_(OH)_2_], CEC=0 mmol/100g

| pyrophyllite_unit_cell | Unit cell (Lee and Guggenheim, 1981) [4] |
| --- | --- |
| pyrophyllite30_cell | Supercell (5x3x2) |
| pyrophyllite15_single_layer | Single layer (5x3x1) in rectangular cell |

References related to clay minerals:

[1]	Heinz, H.; Castelijns, H. J.; Suter, U. W. J. Am. Chem. Soc. 2003, 125, 9500-9510. Basic concepts for the force field for mica.

[2]	Heinz, H.; Suter, U. W. Angew. Chem. Int. Ed. 2004, 43, 2239-2243. Distribution of charge defects according to solid-state NMR data.

[3]	Heinz, H.; Suter, U. W. J. Phys. Chem. B 2004, 108, 18341-18352. An reliable method to assign atomic charges and examples.

[4]	Heinz, H.; Koerner, H.; Anderson, K. L.; Vaia, R. A.; Farmer, B. L. Chem. Mater. 2005, 17, 5658-5669. Complete force field for the 3 minerals, accurate reproduction of surface energies, unit cell parameters, vibrational constants, and compatibility with biomolecular force fields.

**3.2. FCC Metals**

This folder contains unit cells (Fm3m) of the elemental metals Ag, Al, Au, Cu, Ni, Pd, Pb, and Pt and 24 specific smallest cells to prepare periodic models of {111}, {100}, and {110} surfaces of these metals in .car/.mdf format. The respective surfaces are oriented perpendicular to the z direction.

Reference related to FCC Metals:

[1]	Heinz, H.; Vaia, R. A.; Farmer, B. L.; Naik, R. R. J. Phys. Chem. 2008, 112, 17281-17290. Parameters and validation.

[2]	Heinz, H.; Farmer, B. L.; Pandey, R. B.; Slocik, J. M.; Patnaik, S. S.; Pachter, R.; Naik, R. R. J. Am. Chem. Soc. 2009, 131, 9704-9714. Identification of peptide binding mechanisms.

[3]	Heinz, H.; Jha, K. C.; Luettmer-Strathmann, J.; Farmer, B. L.; Naik, R. R. J. R. Soc. Interface 2011, 8, 220-232. Evaluation of induced charges and water interfaces.

[4]	Binding of amino acids (Soft Matter 2011, 7, 2113), prediction of peptide binding to Pd and catalytic activity (JACS 2011, 133, 12346; PCCP 2013, 15, 5488; ACS Nano 2015), understanding nanocrystal growth and shape control (Nano Lett 2013, 13, 840; ACS Nano 2014, 8, 6934; Adv Funct. Mater. 2015, 25, 1374).

**3.3. Silica**

The models encompass unit silica unit cells and twenty different surface models that span the full range of surface chemistry and pH (see Table 1). The format of the models includes .car/.mdf for Materials Studio, Forcite and Discover programs, .lammps05 for LAMMPS, as well as .pdb/.psf for NAMD, CHARMM, and AMBER programs. Atomic coordinates of the surface models are coarsely energy-minimized. A few steps initial minimization (e.g. 50) before molecular dynamics are recommended to adjust atom positions to new interfacial environments.

Silica surfaces with customized surface topology, degree of ionization, and porosity can be prepared from the bulk and surface models provided. Correct assignment of stoichiometry, atom connectivity, atomic charges, and force field types is essential for customized models (the net charge should be 0.0000e).

**Table 1.** Guide towards surface models for a given silica substrate [1].

| Type of silica substrate | 1. Area density of silanol groups | 2. Ionization to  (SiO^–^ M^+^)^a^ | 3. Surface topography |
| --- | --- | --- | --- |
| Quartz surfaces, silica nanoparticles >200 nm size, silica at pH > 9 | Q^2^ and Q^2^/Q^3^  (9.4 to 4.7 per nm^2^) | pH 2: ~0 per nm^2^  pH 5: ~0.5 per nm^2^  pH 7: ~1.0 per nm^2^  pH ≥9: ~1.5 per nm^2^ | Substrate-specific:  smooth,  rough,  porous |
| Most silica glasses, porous silica, silica nanoparticles <200 nm size | Q^3^  (4.7 per nm^2^) | pH 3: ~0 per nm^2^ (0%)  pH 5: ~0.3 per nm^2^ (6%)  pH 7: ~0.6 per nm^2^ (13%)  pH ≥9: ~0.9 per nm^2^ (20%) |  |
| Silica surfaces and nanoparticles annealed at 200-1000 °C | Q^3^/Q^4^ and Q^4^  (4.7 to 0 per nm^2^) | pH 4: ~0 per nm^2^  pH 7: 0-0.6 per nm^2^ depending on Q^3^ content |  |

^a^ Physiological conditions with an ionic strength *I* ~ 0.1-0.3 M of sodium ions (M=Na) are assumed. Ionization at a given pH for different ionic strength *I* and other cations must be reevaluated according to reference data (see [1]).

**3.3.1. Bulk Silica**

Model formula: SiO_2_

| a_cristobalite_unit_cell | Unit cell of α-cristobalite to create Q3 silica surfaces on the {20-2} plane with an area density of (SiOH + SiO^−^ Na^+^) groups of 4.7 per nm^2^ |
| --- | --- |
| a_cristobalite_20m2_cell | Smallest cell of α-cristobalite in which the {20-2} plane is perpendicular to the z direction |
| a_quartz_unit_cell | Unit cell of α-quartz to create Q2 silica surfaces on the {001} plane with an area density of (SiOH + SiO^−^ Na^+^) groups of 9.4 per nm^2^ |
| a_quartz_001_rectangular_cell | Smallest cell of α-quartz cell with rectangular repeat and in which the {001} plane is perpendicular to the z direction |

**3.3.2. Silica Surfaces**

A suitable model corresponding to silica surface type and pH can be chosen using Table 1 [1].

Model formula: [SiO_2_]_n_ with surface termination by (SiOH)_x_ and (SiONa)_y_ groups; the ionized percentage is $\frac{y}{x+y}\cdot100\%$

| silica_Q2_9_4OH_0pct_ion | Q2 surface with 9.4 silanol groups per nm^2^ prepared from a (7x4x3) supercell of the α-quartz 001 rectangular cell by cleavage and hydration of the {001} plane, 0.0 SiO^-^Na^+^ per nm^2^ (pzc) |
| --- | --- |
| silica_Q2_9_4OH_9pct_ion | 9.4 SiO(H,Na) per nm^2^, 0.84 SiO^-^Na^+^ per nm^2^ |
| silica_Q2_9_4OH_18pct_ion | 9.4 SiO(H,Na) per nm^2^, 1.69 SiO^-^Na^+^ per nm^2^ |
| silica_Q2_Q3_6_9OH_0pct_ion | Mixed Q2/Q3 surface with 6.9 silanol groups per nm^2^ prepared from the Q3 surface by hydrolysis of surface silanol groups, 0.0 SiO^-^Na^+^ per nm^2^ (pzc) |
| silica_Q2_Q3_6_9OH_9pct_ion | 6.9 SiO(H,Na) per nm^2^, 0.60 SiO^-^Na^+^ per nm^2^ |
| silica_Q2_Q3_6_9OH_18pct_ion | 6.9 SiO(H,Na) per nm^2^, 1.29 SiO^-^Na^+^ per nm^2^ |
| silica_Q3_4_7OH_0pct_ion | Q3 surface with 4.7 silanol groups per nm^2^ prepared from a (4x7x2) supercell of the α-cristobalite 20m2 cell with cleavage and hydration of the {20-2} plane, 0.0 SiO^-^Na^+^ per nm^2^ (pzc) |
| silica_Q3_4_7OH_5pct_ion | 4.7 SiO(H,Na) per nm^2^, 0.25 SiO^-^Na^+^ per nm^2^ |
| silica_Q3_4_7OH_9pct_ion | 4.7 SiO(H,Na) per nm^2^, 0.42 SiO^-^Na^+^ per nm^2^ |
| silica_Q3_4_7OH_14pct_ion | 4.7 SiO(H,Na) per nm^2^, 0.67 SiO^-^Na^+^ per nm^2^ |
| silica_Q3_4_7OH_18pct_ion | 4.7 SiO(H,Na) per nm^2^, 0.84 SiO^-^Na^+^ per nm^2^ |
| silica_Q3_4_7OH_27pct_ion | 4.7 SiO(H,Na) per nm^2^, 1.26 SiO^-^Na^+^ per nm^2^ |
| silica_Q3_4_7OH_50pct_ion | 4.7 SiO(H,Na) per nm^2^, 2.35 SiO^-^Na^+^ per nm^2^ |
| silica_Q3_amorph_4_7OH_0pct_ion | Amorphous Q3 surface with 4.7 silanol groups per nm^2^ that contains some Q2 and Q4 environments (adapted from Materials Studio), the area of this corrugated surface is ~5% larger than a flat surface, 0.0 SiO^-^Na^+^ per nm^2^ (pzc) |
| silica_Q3_amorph_4_7OH_9pct_ion | 4.7 SiO(H,Na) per nm^2^, 0.45 SiO^-^Na^+^ per nm^2^ |
| silica_Q3_amorph_4_7OH_18pct_ion | 4.7 SiO(H,Na) per nm^2^, 0.85 SiO^-^Na^+^ per nm^2^ |
| silica_Q3_Q4_2_4OH_0pct_ion | Mixed Q3/Q4 surface with 2.4 silanol groups per nm^2^ prepared from the Q3 surface by partial condensation of surface silanol groups, 0.0 SiO^-^Na^+^ per nm^2^ (pzc) |
| silica_Q3_Q4_2_4OH_15pct_ion | 2.4 SiO(H,Na) per nm^2^, 0.34 SiO^-^Na^+^ per nm^2^ |
| silica_Q3_Q4_2_4OH_30pct_ion | 2.4 SiO(H,Na) per nm^2^, 0.68 SiO^-^Na^+^ per nm^2^ |
| silica_Q4_0_0OH | Q4 surface with 0.0 silanol groups per nm^2^ prepared from the Q3 surface by complete condensation of surface silanol groups and energy minimization (some Si‒O bonds are 10% stretched) |

References related to silica:

[1]	Emami, F. S., Puddu, V. et al. Chem. Mater. 2014, 26, DOI: 10.1021/cm500365c. Full discussion of force field parameters, models, and validation. A separate publication specifically illustrates the accuracy of computed binding free energies of different peptides as a function of pH and particle type (in progress).

[2]	Patwardhan, S. V.; Emami, F. S.; Berry, R. J.; Jones, S. E.; Naik, R. R.; Deschaume, O.; Heinz, H.; Perry, C. C. J. Am. Chem. Soc. 2012, 134, 6244-6256. Initial data showing the importance of silica surface characteristics, water interfaces, and the mechanism of selective biomolecular adsorption.

**3.4. Hydroxyapatite**

Models include the unit cell of hydroxyapatite (HAP) unit cell and twelve surface models for the common {001}, {010}, {020}, and {101} facets in .car/.mdf format that cover the range of pH values from 5 to 14 [1, 2]. The respective surfaces are oriented perpendicular to the z direction. Also, models of two HAP nanocrystals of 3 and 5 nm size at high and low pH, as well as models of the formula units of Na_2_HPO_4_ and NaH_2_PO_4_ in phosphate buffers are provided. Atomic coordinates of the surface models are coarsely energy-minimized, however, brief energy minimization (e.g. 50 steps) is recommended to adjust atom positions in new interfacial environments before molecular dynamics simulation.

Apatite surfaces with customized surface topology, pH, and defect sites can be prepared from the bulk and surface models provided, paying attention to the assignment of surface chemistry, stoichiometry, atom connectivity, atomic charges, and force field types (the net charge should be 0.0000e).

**3.4.1. Bulk Hydroxyapatite**

Model formula: Ca_5_(PO_4_)_3_(OH)

| hap_unit_cell | Unit cell of hydroxyapatite (Hughes, 2002, [3]) |
| --- | --- |
| hap_rectangular_cell | Smallest cell of hydroxyapatite with rectangular repeat, and in which the {001} plane is perpendicular to the z direction |

**3.4.2. Hydroxyapatite Surfaces**

Model formula:

pH >14: Ca_5_(PO_4_)_3_(OH)
pH ~10: Ca_5_(PO_4_)_3_(OH) with HPO_4_^2-^ on the surface and removal of superficial Ca(OH)_2_
pH ~ 5: Ca_5_(PO_4_)_3_(OH) with H_2_PO_4_^-^ on the surface and removal of superficial Ca(OH)_2_

Use mixed PO_4_^3-^, HPO_4_^2-^, H_2_PO_4_^-^, and H_3_PO_4_ termination to customize other pH values [1,2]

| hap_001_pH5_H2PO4 | H_2_PO_4_^-^ terminated {001} surface prepared from a (4x2x4) supercell of the hydroxyapatite rectangular cell by cleavage and hydration of the {001} plane, corresponds to pH~5.0 |
| --- | --- |
| hap_001_pH10_HPO4 | Same with HPO_4_^-^ termination, corresponds to pH~10.0 |
| hap_001_pH14_PO4 | Same for the neat {001} cleavage plane without hydration, corresponds to pH>14 |
| hap_010_pH5_H2PO4 | H_2_PO_4_^-^ terminated {010} surface prepared from a (4x2x5) supercell of the hydroxyapatite rectangular cell by cleavage and hydration of the {010} plane, corresponds to pH~5.0 |
| hap_010_pH10_HPO4 | Same with HPO_4_^-^ termination, corresponds to pH~10.0 |
| hap_010_pH14_PO4 | Same for the neat {010} cleavage plane without hydration, corresponds to pH>14 |
| hap_020_pH5_H2PO4 | H_2_PO_4_^-^ terminated {020} surface prepared from a (4x2x5) supercell of the hydroxyapatite rectangular cell by cleavage and hydration of the {020} plane, corresponds to pH~5.0 |
| hap_020_pH10_HPO4 | Same with HPO_4_^-^ termination, corresponds to pH~10.0 |
| hap_020_pH14_PO4 | Same for the neat {020} cleavage plane without hydration, corresponds to pH>14 |
| hap_101_pH5_H2PO4 | H_2_PO_4_^-^ terminated {101} surface prepared from a supercell of a customized hydroxyapatite rectangular cell that exhibits a {101} surface in the z direction; followed by cleavage and hydration of the {101} plane, corresponds to pH~5.0 |
| hap_101_pH10_HPO4 | Same with HPO_4_^-^ termination, corresponds to pH~10.0 |
| hap_101_pH14_PO4 | Same for the neat {101} cleavage plane without hydration, corresponds to pH>14 |
| hap_nanorod_3nm_pH5_H2PO4 | Draft of a hydroxyapatite nanocrystal bounded by {010} and {001} surfaces with H_2_PO_4_^-^ termination of 3 nm size, corresponds to pH~5.0 |
| hap_nanorod_3nm_pH14_PO4 | Same with neat, non-hydrated cleavage planes, corresponding to pH>14 |
| hap_nanorod_5nm_pH5_H2PO4 | Draft of a hydroxyapatite nanocrystal bounded by {010} and {001} surfaces with H_2_PO_4_^-^ termination of 5 nm size, corresponds to pH~5.0 |
| hap_nanorod_5nm_pH14_PO4 | Same with neat, non-hydrated cleavage planes, corresponding to pH>14 |
| Na2HPO4 | Formula unit of Na_2_HPO_4_ |
| NaH2PO4 | Formula unit of NaH_2_PO_4_ |

References related to Hydroxyapatite:

[1]	Lin, T. Z. *Force Field Parameters and Atomistic Surface Models for Hydroxyapatite and Analysis of Biomolecular Adsorption at Aqueous Interfaces*; University of Akron, PhD thesis, 2013, <http://rave.ohiolink.edu/etdc/view?acc_num=akron1365511604>. Describes force field parameters, surface models, applications to peptides and drug molecules. Separate publications in progress.

[2]	Heinz, H. J. Phys. Cond. Mat. 2014, 26, 244105. Publications with more details to follow.

[3]	Hughes, J. M.; Rakovan, J. Rev. Mineral. Geochem. 2002, 48, 1-12. This reference contains the unit cell parameters for apatites (OH, F, Cl). Separate publications in progress.

**3.5. Cement Minerals**

This folder contains the unit cells, surfaces, and modified surfaces of minerals in cement in .car/.mdf format.

| **Tricalcium aluminate (C3A)** | Model formula: Ca_3_Al_2_O_6_ |
| --- | --- |
| c3a_unit_cell | Unit cell of C3A according to Mondal and Jeffery, 1975 |
| c3a_unit_cell_minimized | Same with optimized cell parameters for use |
| c3a_010_surface_together | Thick slab of the corrugated {010} surface from a 2x4x2 super cell after relaxation |
| c3a_010_surface_separated | Two thinner slabs of the corrugated {010} surface corresponding to 2x2x2 super cells upon equilibrium cleavage and relaxation |
| c3a_010_surface_hydrated_small | {010} surface from a 2x2x1 super cell upon initial hydration of first and second aluminate layer |
| c3a_010_surface_hydrated_large | {010} surface from a 2x2x3 super cell upon initial hydration of first and second aluminate layer |
| c3a_010_surface_hydrated_h2o | {010} surface from 2x2x2 super cell upon initial hydration of first and second aluminate layer immersed in water |
|  |  |
| **Tricalcium silicate (C3S)** | Model formula: Ca_3_SiO_5_ |
| c3s_unit_cell | Unit cell of C3S, M3 polymorph, according to Mumme, 1995 |
| c3s_unit_cell_minimized | Same with optimized cell parameters |
| c3s_rectangular_cell_minimized | Same with rectangular cell parameters |
| c3s_040_surface | Cleaved {040} surface with equilibrium distribution of Ca^2+^ cations (based on a 2x3x1 supercell of the rectangular cell);  the {040} cleavage plane is of lowest energy |
| c3s_040_surface_hydroxylated | Hydroxylated {040} surface using the previous model;  represents the initial step in C3S hydration |
|  |  |
| **Ettringite** | Model formula: Ca_6_Al_2_(SO_4_)_3_(OH)_12_ · 27 H_2_O |
| ettr_unit_cell | Unit cell according to Moore, 1969, in .car/.mdf format |
| ettr_rectangular_cell | Same as above with rectangular repeat |
| ettr_200_surface | Model of the equilibrium {200} surface using a 1x4x2 supercell of the rectangular cell; the {200} plane is of lowest cleavage energy |
| ettr_008_surface | Model of the equilibrium {008} surface using a 2x4x1 supercell of the rectangular cell |
|  |  |
| **Monosulfate** | Model formula: Ca_4_Al_2_(SO_4_)(OH)_12_ · 6 H_2_O |
| monosulfate_cell_221 | 2x2x1 super cell according to Allmann, 1968, in .car/.mdf format;  multiple cells are needed to accommodate average occupancies |
| monosulfate_rectangular_cell_421 | Same as above with rectangular repeat |
| monosulfate_002_surface | Model of the equilibrium {002} surface using a 1x1x(2/3) supercell of the rectangular cell |
|  |  |
| **Tobermorite 11 Å** | Model formula: Ca_4_Si_6_O_15_(OH)_2_ · 5 H_2_O |
| tob11_unit_cell | Unit cell according to Merlino, 1999, in .car/.mdf format |
| tob11_rectangular_cell | Same as above with a rectangular repeat |
| tob11_100_surface | Model of the equilibrium {100} surface using a 3x3x1 supercell of the rectangular cell |
| tob11_004_surface | Model of the equilibrium {004} surface using a 2x3x1.5 supercell of the rectangular cell |
|  |  |
| **Tobermorite 14 Å** | Model formula: Ca_5_Si_6_O_16_(OH)_2_· 7 H_2_O |
| tob14_avg_cell | Unit cell according to Merlino, 1999, in .car/.mdf format;  average statistical occupancies included |
| tob14_avg_rectangular_cell | Same as above with a rectangular repeat |
| tob14_100_surface | Model of the equilibrium {100} surface using a 2x3x1 supercell of the rectangular cell |
| tob14_001_surface | Model of the equilibrium {001} surface using a 2x3x1 supercell of the rectangular cell |
| tob14_004_surface | Model of the equilibrium {004} surface using a 2x3x1 supercell of the rectangular cell |

Large model systems of all surfaces, interfaces with water, and with organic molecules have been extensively tested for all compounds.

References related to cement minerals:

[1]	Heinz, H.; Suter, U. W. J. Phys. Chem. B 2004, 108, 18341-18352. Discussion of atomic charges.

[2]	Heinz, H.; Lin, T.-J., Mishra, R. K.; Emami, F. S. Langmuir 2013, 29, 1754-1765. First complete publication of parameters for all minerals.

[3]	Mishra, R. K.; Flatt, R. J.; Heinz, H. J. Phys. Chem. C 2013, 117, 10417-10432. Details on C_3_S.

[4]	Mishra, R. K.; Fernandez-Carrasco, L.; Flatt, R. J.; Heinz, H. Dalton Transactions 2014, DOI:10.1039/C4DT00438H. Details on C_3_A. Further publications with more details on the other minerals are in progress.

**3.6. Ca Sulfates**

This folder contains unit cells of anhydrite (Hawthorne, 1975), calcium sulfate hemihydrate (Bezou, 1995) and gypsum (space group A2/a, Pedersen, 1982) in .car/.mdf format. Superstructures and cleavage planes can be easily created.

Model formulas: CaSO_4_, CaSO_4_ · 0.5 H_2_O, and CaSO_4_ · 2 H_2_O

References related to calcium sulfates:

[1]	Heinz, H. Reports internal to Sika AG (2007-2009).

[2]	Mishra, R. K. PhD Dissertation, University of Akron, 2012. Separate publications in progress.

**3.7. PEO**

| **Polyethylene oxide** | Model formula −(CH_2_−CH_2_−O−)_n_ |
| --- | --- |
| peo_cell | Unit cell of polyethylene oxide from Takahashi, 1973, in .car./.mdf format;  models of polymer chains in solution reproduce the approximate radius of gyration, hydration energy, and gauche effect |
| Peo46_h2o4394_pcff | polyethylene oxide of length n = 46, molecular weight ~2000 g/mol, in solution with 4394 water molecules |

The parameters for PEO have not yet been separately published.

**4. History of Versions**

The origin of the INTERFACE force field is consistent parameters for muscovite mica and clay minerals (2002-2005). Then, extensions for cement minerals were added (2003-2004, proprietary to Sika AG until 2012), followed by fcc metals, sulfates, silica, PEO, apatite, and compounds in progress. The first release on a web site was PCFF_PHYLLOSILICATES. Table 2 depicts the history of versions with approximate dates. A log of specific improvements and updates for each version of the INTERFACE force field is given separately.

**Table 2.** Origin of the INTERFACE force field and prior versions.

| Release | Date | Brief description |
| --- | --- | --- |
| INTERFACE 1.3 | 2014/04 | All parameters, multiple platforms, extensive surface model database |
| INTERFACE 1.2 | 2013/04 |  |
| INTERFACE 1.0/1.1 | 2013/01 |  |
| CHARMM-METAL | 2012/08- 2010/03 | Extension of CHARMM for fcc metals |
| FF_LS_METAL | 2013/01- 2008/06 | Extension of PCFF and CVFF for clay minerals and fcc metals |
| FF_PHYLLOSILICATES (PCFF-PHYLLOSILICATES, CVFF-PHYLLOSILICATES) | 2010/07-2008/03 | Extension of PCFF and CVFF for clay minerals |
| PCFF_LS_CEMENT | (Proprietary 2012-2003 until INTERFACE) | Extensions of PCFF for layered silicates, cement minerals, and other compounds |
