# Supplementary material for: Devising Bone Molecular Models at the Nanoscale: From Usual Mineralized Collagen Fibrils to the First Bone Fibers Including Hydroxyapatite in the Extra-Fibrillar Volume
Source: Materials (Basel). 2022 Mar 19;15(6):2274. doi: 10.3390/ma15062274 (PMC8955169; doi:10.3390/ma15062274)
Supplement: Supplementary file 1 [file materials-15-02274-s001.zip › Supplementary_Materials/3-Bone_Fiber/HA/INTERFACE_MD/interface_ff_1_5.zip!/INTERFACE_FF_1_5/MODEL_DATABASE/SILICA/Description_of_silica_models.docx]

**Description of Silica Models**

Consult Table 1 at the end to choose a model corresponding to silica surface type and pH [1]

| **Models of bulk silica**  SiO_2_ | **Description** |
| --- | --- |
| a_cristobalite_unit_cell | Unit cell of α-cristobalite to create Q3 silica surfaces on the {20-2} plane with an area density of (SiOH + SiO^−^ Na^+^) groups of 4.7 per nm^2^ |
| a_cristobalite_20m2_cell | Smallest cell of α-cristobalite in which the {20-2} plane is perpendicular to the z direction |
| a_quartz_unit_cell | Unit cell of α-quartz to create Q2 silica surfaces on the {001} plane with an area density of (SiOH + SiO^−^ Na^+^) groups of 9.4 per nm^2^ |
| a_quartz_001_rectangular_cell | Smallest cell of α-quartz cell with rectangular repeat and in which the {001} plane is perpendicular to the z direction |
| **Models of silica surfaces**  [SiO_2_]_n_ with surface termination by (SiOH)_x_ and (SiONa)_y_ groups | **Description** |
| silica_Q2_9_4OH_0pct_ion | Q2 surface with 9.4 silanol groups per nm^2^ prepared from a (7x4x3) supercell of the α-quartz 001 rectangular cell by cleavage and hydration of the {001} plane, 0.0 SiO^-^Na^+^ per nm^2^ (pzc) |
| silica_Q2_9_4OH_9pct_ion | 9.4 SiO(H,Na) per nm^2^, 0.84 SiO^-^Na^+^ per nm^2^ |
| silica_Q2_9_4OH_18pct_ion | 9.4 SiO(H,Na) per nm^2^, 1.69 SiO^-^Na^+^ per nm^2^ |
| silica_Q2_Q3_6_9OH_0pct_ion | Mixed Q2/Q3 surface with 6.9 silanol groups per nm^2^ prepared from the Q3 surface by hydrolysis of surface silanol groups, 0.0 SiO^-^Na^+^ per nm^2^ (pzc) |
| silica_Q2_Q3_6_9OH_9pct_ion | 6.9 SiO(H,Na) per nm^2^, 0.60 SiO^-^Na^+^ per nm^2^ |
| silica_Q2_Q3_6_9OH_18pct_ion | 6.9 SiO(H,Na) per nm^2^, 1.29 SiO^-^Na^+^ per nm^2^ |
| silica_Q3_4_7OH_0pct_ion | Q3 surface with 4.7 silanol groups per nm^2^ prepared from a (4x7x2) supercell of the α-cristobalite 20m2 cell with cleavage and hydration of the {20-2} plane, 0.0 SiO^-^Na^+^ per nm^2^ (pzc) |
| silica_Q3_4_7OH_5pct_ion | 4.7 SiO(H,Na) per nm^2^, 0.25 SiO^-^Na^+^ per nm^2^ |
| silica_Q3_4_7OH_9pct_ion | 4.7 SiO(H,Na) per nm^2^, 0.42 SiO^-^Na^+^ per nm^2^ |
| silica_Q3_4_7OH_14pct_ion | 4.7 SiO(H,Na) per nm^2^, 0.67 SiO^-^Na^+^ per nm^2^ |
| silica_Q3_4_7OH_18pct_ion | 4.7 SiO(H,Na) per nm^2^, 0.84 SiO^-^Na^+^ per nm^2^ |
| silica_Q3_4_7OH_27pct_ion | 4.7 SiO(H,Na) per nm^2^, 1.26 SiO^-^Na^+^ per nm^2^ |
| silica_Q3_4_7OH_50pct_ion | 4.7 SiO(H,Na) per nm^2^, 2.35 SiO^-^Na^+^ per nm^2^ |
| silica_Q3_amorph_4_7OH_0pct_ion | Amorphous Q3 surface with 4.7 silanol groups per nm^2^ that contains some Q2 and Q4 environments (adapted from Materials Studio), the area of this corrugated surface is ~5% larger than a flat surface, 0.0 SiO^-^Na^+^ per nm^2^ (pzc) |
| silica_Q3_amorph_4_7OH_9pct_ion | 4.7 SiO(H,Na) per nm^2^, 0.45 SiO^-^Na^+^ per nm^2^ |
| silica_Q3_amorph_4_7OH_18pct_ion | 4.7 SiO(H,Na) per nm^2^, 0.85 SiO^-^Na^+^ per nm^2^ |
| silica_Q3_Q4_2_4OH_0pct_ion | Mixed Q3/Q4 surface with 2.4 silanol groups per nm^2^ prepared from the Q3 surface by partial condensation of surface silanol groups, 0.0 SiO^-^Na^+^ per nm^2^ (pzc) |
| silica_Q3_Q4_2_4OH_15pct_ion | 2.4 SiO(H,Na) per nm^2^, 0.34 SiO^-^Na^+^ per nm^2^ |
| silica_Q3_Q4_2_4OH_30pct_ion | 2.4 SiO(H,Na) per nm^2^, 0.68 SiO^-^Na^+^ per nm^2^ |
| silica_Q4_0_0OH | Q4 surface with 0.0 silanol groups per nm^2^ prepared from the Q3 surface by complete condensation of surface silanol groups and energy minimization (some Si‒O bonds are 10% stretched) |

Notes:

(1) The models contain appropriate atom types and atomic charges. They are ready for simulations using codes such as Forcite, Discover (.car/.mdf), LAMMPS (.lammps05), NAMD, AMBER (.pdb/.psf), and others.

(2) The surface models contain reasonable initial atomic coordinates and are coarsely energy-minimized. A few steps initial minimization (e.g. 50) before molecular dynamics are recommended to adjust atom positions to new interfacial environments.

(3) Silica surfaces with customized degree of ionization and surface topology, including porous structures, can be prepared from the given bulk and surface models. Correct assignment of stoichiometry, atom connectivity, atomic charges, and force field types is essential (the net charge should be 0.0000e).

**Table 1. Guide towards Surface Models for a Given Silica Substrate [1]**

| Type of silica substrate | 1. Area density of silanol groups | 2. Ionization to  (SiO^–^ M^+^)^a^ | 3. Surface topography |
| --- | --- | --- | --- |
| Quartz surfaces, silica nanoparticles >200 nm size, silica at pH > 9 | Q^2^ and Q^2^/Q^3^  (9.4 to 4.7 per nm^2^) | pH 2: ~0 per nm^2^  pH 5: ~0.5 per nm^2^  pH 7: ~1.0 per nm^2^  pH ≥9: ~1.5 per nm^2^ | Substrate-specific:  smooth,  rough,  porous |
| Most silica glasses, porous silica, silica nanoparticles <200 nm size | Q^3^  (4.7 per nm^2^) | pH 3: ~0 per nm^2^ (0%)  pH 5: ~0.3 per nm^2^ (6%)  pH 7: ~0.6 per nm^2^ (13%)  pH ≥9: ~0.9 per nm^2^ (20%) |  |
| Silica surfaces and nanoparticles annealed at 200-1000 °C | Q^3^/Q^4^ and Q^4^  (4.7 to 0 per nm^2^) | pH 4: ~0 per nm^2^  pH 7: 0-0.6 per nm^2^ depending on Q^3^ content |  |

^a^ Physiological conditions with an ionic strength *I* ~ 0.1-0.3 M of sodium ions (M=Na) are assumed. Ionization at a given pH for different ionic strength *I* and other cations must be reevaluated according to reference data (see text).

References:

[1]	Emami, F. S. et al. Chem. Mater. 2014, 26, DOI: 10.1021/cm500365c.

[2]	Patwardhan, S. V.; Emami, F. S.; Berry, R. J.; Jones, S. E.; Naik, R. R.; Deschaume, O.;	Heinz, H.; Perry, C. C. J. Am. Chem. Soc. 2012, 134, 6244-6256.
