# Supplementary material for: Devising Bone Molecular Models at the Nanoscale: From Usual Mineralized Collagen Fibrils to the First Bone Fibers Including Hydroxyapatite in the Extra-Fibrillar Volume
Source: Materials (Basel). 2022 Mar 19;15(6):2274. doi: 10.3390/ma15062274 (PMC8955169; doi:10.3390/ma15062274)
Supplement: Supplementary file 1 [file materials-15-02274-s001.zip › Supplementary_Materials/Instructions.pdf]

The files and scripts within this folder belong to the Supplementary Material of the paper:

Devising Bone Molecular Models at the Nanoscale: From Usual Mineralized Collagen Fibrils to the First Bone Fibers including Hydroxyapatite in the Extra-Fibrillar Volume

Amadeus C. S. de Alcântara, Levi C. Felix, Douglas S. Galvão, Paulo Sollero, Munir S. Skaf

Materials MDPI 2022

The paper, together with these files, aims to provide a detailed prescription on how to devise all-atom bone molecular models at the nanoscale that include hydroxyapatite in the extra-fibrillar volume, resembling fibers in bones.

Tips:

- To understand the details of the modeling process, please read the paper (including Appendices) and the README files in each folder thoroughly.

- Follow the folders in increasing order, i.e., 0-COL1_Modeller first, and 3-Bone_Filer at last.

- Each README file informs the INPUT/OUTPUT of each step and describes how that specific step can be performed/reproduced.

The figure below shows a flowchart of the modeling process of our Bone Fiber model.

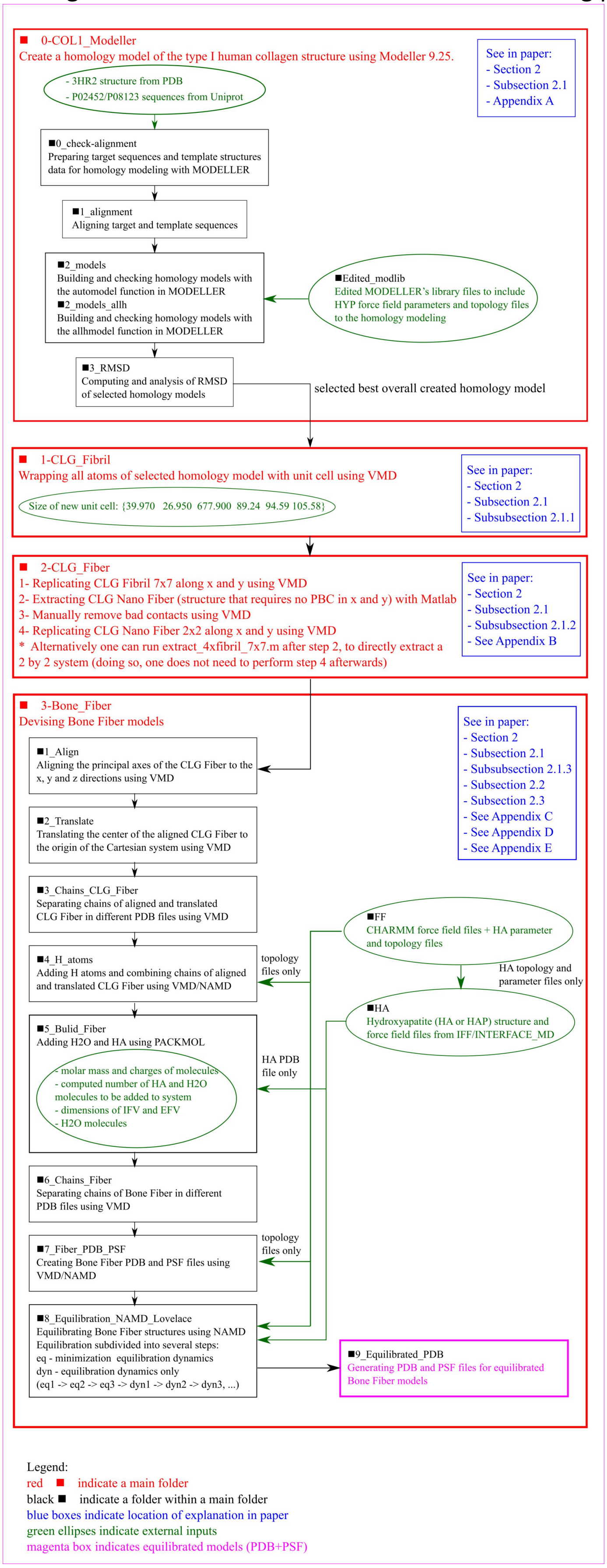

Content of each folder:

- 0-COL1_Modeller

See Section 2. Devising Bone at the Nanoscale
See Subsection 2.1. Devising the Simulation Box
See Appendix A – Building Homology Models of Human Type-I Collagen using MODELLER 9.25

Files used to create a homology model of the type I human collagen structure using Modeller 9.25.
Descriptive README files.

  - 0_check-alignment
  Preparing target sequences and template structures data for homology modeling with MODELLER

  - 1_alignment
  Aligning target and template sequences

  - 2_models
  Building and checking homology models with the automodel function in MODELLER

  - 2_models_allh
  Building and checking homology models with the allhmodel function in MODELLER

  - 3_RMSD
  Computing and analysis of RMSD of selected homology models

  - Edited_modlib
  Edited MODELLER's library files to include HYP force field parameters and topology files to the homology modeling

See https://salilab.org/modeller/tutorial/ for further details on homology/comparative modeling of protein three-dimensional structures with MODELLER.

- 1-CLG_Fibril

See Section 2. Devising Bone at the Nanoscale
See Subsection 2.1. Devising the Simulation Box
See Subsubsection 2.1.1. CLG Fibril

Devising CLG Fibril - Compressing/Wrapping homology model in Unit Cell (UC)

Files used to devise CLG Fibril model, i.e., to wrap all atoms in predefined unit cell using VMD.
Descriptive README file.

- 2-CLG_Fiber

See Section 2. Devising Bone at the Nanoscale
See Subsection 2.1. Devising the Simulation Box
See Subsubsection 2.1.2. CLG Fiber
See Appendix B – CLG Fiber

Devising the CLG Fiber - Replicating CLG Fibril, Extracting CLG Nano Fiber, and Replicating CLG Nano Fiber

Files used to devise CLG NanoFiber model.
 Files used to replicate CLG Fibril along x and y directions using VMD.
 Files used to extract from a structure that requires no PBC in x and y using Matlab.
Files used to devise CLG Fiber model.
 Files used to replicate CLG NanoFiber along x and y directions using VMD.
Descriptive README file.

- 3-Bone_Fiber

See Section 2. Devising Bone at the Nanoscale
See Subsection 2.1. Devising the Simulation Box
See Subsubsection 2.1.3. Bone Fiber
See Subsection 2.3. Minimization and Equilibration
See Appendix C – Bone Fiber

Files used to devise Bone Fiber models.

  - 1_Align
    Aligning the principal axes of the model to the x, y and z directions using VMD

  - 2_Translate
    Translating the center of the model to the origin of the Cartesian system using VMD

  - 3_Chains_CLG_Fiber
    Separating chains of aligned and translated CLG Fiber in different PDB files using VMD

  - 4_H_atoms

Adding H atoms to aligned and translated CLG Fiber using VMD/NAMD

- 5_Bulid_Fiber
See Appendix D – Mass fraction calculation
Adding H2O and HA using PACKMOL

- 6_Chains_Fiber
Separating chains of Bone Fiber in different PDB files using VMD

- 7_Fiber_PDB_PSF
Creating Bone Fiber PDB and PSF files using VMD/NAMD

- 8_Equilibration_NAMD_Lovelace
Equilibrating Bone Fiber structures using NAMD

- 9_Equilibrated_PDB
Generating PDB and PSF files for equilibrated Bone Fiber models

- FF
See Subsection 2.2. Force Fields
CHARMM force field files + HA parameter and topology files

- HA
See Appendix E – HA structure and FF files
Hydroxyapatite (HA or HAP) structure and force field files from IFF/INTERFACE_MD

Descriptive README files.

Written by Amadeus Alcântara
amadeus.alcantara@gmail.com

CITE:

Devising Bone Molecular Models at the Nanoscale: From Usual Mineralized Collagen Fibrils to the First Bone Fibers including Hydroxyapatite in the Extra-Fibrillar Volume

Amadeus C. S. de Alcântara, Levi C. Felix, Douglas S. Galvão, Paulo Sollero, Munir S. Skaf

Materials MDPI 2022
